# Dormancy dynamics in Japanese plum: transcriptomic responses to variable climatic conditions and chilling requirements

**DOI:** 10.1007/s00425-026-05104-w

**Published:** 2026-08-01

**Authors:** Sara Herrera, José Ignacio Hormaza, Guillem Ylla, Javier Rodrigo, Jorge Lora

**Affiliations:** 1 Estación Experimental de Aula Dei (EEAD-CSIC), Consejo Superior de Investigaciones Científicas, Zaragoza, Spain; 2 Laboratorio de Climatología y Servicios Climáticos (LCSC), CSIC–Universidad de Zaragoza, Zaragoza, Spain; 3 Departamento de Ciencia Vegetal, Centro de Investigación y Tecnología Agroalimentaria de Aragón (CITA), Zaragoza, Spain; 4 Instituto Agroalimentario de Aragón-IA2 (CITA-Universidad de Zaragoza), Zaragoza, Spain; 5 Instituto de Hortofruticultura Subtropical y Mediterránea La Mayora (IHSM La Mayora-CSIC-UMA), Algarrobo-Costa, 29750 Málaga, Spain; 6 Laboratory of Bioinformatics and Genome Biology, Faculty of Biochemistry, Biophysics and Biotechnology, Jagiellonian University, 30-387 Kraków, Poland

**Keywords:** Climatic adaptation, Chill accumulation, Epigenetics, Gene expression, Phenology, Transcriptome

## Abstract

**Main conclusion:**

Climate exerted a stronger influence than dormancy stage on gene expression in Japanese plum, revealing climate- and genotype-specific regulatory patterns underlying dormancy control and climate adaptation.

**Abstract:**

Dormancy progression in temperate fruit trees is highly sensitive to environmental conditions and chilling accumulation. To investigate the regulation of dormancy in Japanese plum (*Prunus salicina* hybrids), we performed a comparative transcriptomic analysis of flower buds from two Japanese plum cultivars with different chilling requirements, "Hiromi Red" (high chill) and "Crimson Glo" (low chill), grown under contrasting climates (semi-arid and Mediterranean subtropical). This study combined phenological observations, quantification of chill and heat requirements, as well as transcriptomic analyses across three key developmental stages: full dormancy (T0), dormancy release (T1), and full recovery (T2). Climate exerted a stronger influence than dormancy stage on gene expression profiles, leading to cultivar- and climate-specific transcriptional responses. Key dormancy-related genes—such as *DAM*, *FT*, and *SAP1*— exhibited differential expression patterns across climates, suggesting potential roles in climatic adaptation. Notably, dormancy phases occurred approximately one month later under Mediterranean subtropical conditions, and were accompanied by a marked reduction in chilling requirements. Expression and phylogenetic analyses revealed that environmental conditions had a stronger effect on the transcriptomic profiles than the progression of dormancy itself, potentially due to epigenetic modulation. These findings provide new insights into the molecular mechanisms underlying dormancy in woody perennial species and offer perspectives for developing cultivars better adapted to changing climatic scenarios.

**Supplementary Information:**

The online version contains supplementary material available at 10.1007/s00425-026-05104-w.

## Introduction

Flowering plants exhibit numerous adaptive features to survive adverse environmental conditions. In temperate woody perennials, vegetative growth typically occurs in spring and summer, while dormancy begins in autumn. During dormancy, visible growth ceases but low metabolic activity is maintained, protecting plants from harsh environmental conditions (Horvath 2009; Cooke et al. 2012). Dormancy is crucial not only for survival but also for ensuring proper fruit production (Rodrigo 2000; Rohde and Bhalerao 2007).

Dormancy is usually differentiated into three phases: paradormancy (late summer), endodormancy (autumn–winter), and ecodormancy (late winter–spring) (Lang et al. 1987). During endodormancy, bud growth remains inhibited even under favorable temperatures until they accumulate sufficient chilling (chilling requirements) to transition to the next phase, ecodormancy. During ecodormancy, bud growth resumes upon fulfillment of enough warmth (heat requirements) (Lang et al. 1987). Global warming threatens this finely tuned process by reducing chill accumulation in many temperate regions, leading to substantial yield losses (Campoy et al. 2011; Luedeling et al. 2011). A lack of sufficient winter chilling modifies spring phenology (Beil et al. 2021), causing reduced and irregular flowering and fruiting, ultimately leading to lower fruit yield (Kovaleski 2024).

Despite over two centuries of research, the regulatory mechanisms of dormancy remain unclear (Fadón et al. 2020a). Agroclimatic requirements—chill requirements during endodormancy and heat requirements during ecodormancy—are cultivar-specific and determine their adaptation to different regions (Fadón et al. 2020b). Given their significant agroclimatic implications, these requirements have been extensively evaluated in cultivars of stone fruit tree species such as plum (Guerrero et al. 2024), apricot (Ruiz et al. 2007; Herrera et al. 2022), and sweet cherry (Fadón et al. 2021). However, the approaches and models used to estimate these agroclimatic requirements have limitations (Luedeling 2012; Fadón et al. 2023), often leading to estimates that cannot be directly applied to regions with different climatic conditions. To address these limitations, recent studies have explored potential biological markers of the transition from endodormancy to ecodormancy, such as male meiosis in apricot (Herrera et al. 2022).

Since the early century, key dormancy-related genes have been identified, including *DORMANCY-ASSOCIATED MADS-box* (*DAM*), first identified in peach (Bielenberg et al. 2004, 2008) and later in other fruit tree species (Quesada-Traver et al. 2022). Recent transcriptomic research has linked gene expression with hormonal, physiological, and metabolic changes during dormancy (Galindo González et al. 2012; Nishitani et al. 2012; Zhuang et al. 2013; Zhang et al. 2018), but most studies have focused on single climatic conditions. Given the challenges derived from global climate change, there is an urgent need for studies assessing dormancy regulation under diverse environmental conditions.

Recent transcriptomic research on *Prunus* species has assessed dormancy regulation in genotypes adapted to different agroclimatic conditions. In sweet cherry, conserved gene expression patterns have been identified, highlighting seven genes predictive of dormancy stages (Vimont et al. 2019). Comparative transcriptomic analyses of apricot and peach cultivars with different chilling requirements have been conducted, analyzing floral buds at key chill-hour intervals (Yu et al. 2020). Subsequent studies have re-analyzed RNA-seq data to identify key dormancy-related genes in sweet cherry, apricot, and peach (Canton et al. 2021), as well as in almond (Calle et al. 2022). Recently, the genome sequence and a comparative transcriptome study of two Japanese plum cultivars with different chilling requirements identified six tandemly arrayed *PsDAM* genes, including *PsDAM6*, which could potentially influence dormancy and chilling requirements (Fang et al. 2022).

To address this gap, this study aims to elucidate the genetic basis of dormancy progression mechanisms in the floral buds of Japanese plum (hybrids of *P. salicina*) focusing on how environmental conditions influence reproductive development. Using two cultivars with different chilling requirements, we analyzed gene expression under contrasting semi-arid and Mediterranean subtropical conditions to assess the impact of climate on dormancy-related gene expressions and identify key genes involved in endodormancy regulation and transition from endodormancy to ecodormancy.

## Material and methods

### Plant material

Shoots and flower buds were collected from two Japanese plum cultivars, "Hiromi Red" and "Crimson Glo" (hybrids of *Prunus salicina* Lindl.), with contrasting late and early flowering dates, respectively. Trees of the same age and grafted on the same rootstock were grown in two locations in Spain: IHSM La Mayora (IM; Algarrobo-Costa, Málaga, 36°45′23.4"N, 4°02′35.9"W, 25 m altitude, hot-summer Mediterranean climate) and Finca La Redonda (La Almunia de Doña Godina, Zaragoza, 41°27′28.6"N, 1°21′33.7"W, 394 m altitude, cold semi-arid climate) (Fig. S1). The hot-summer Mediterranean climate (Csa) of Algarrobo-Costa (Peel et al. 2007), also referred to as a Mediterranean subtropical climate due to its support for the extensive cultivation of subtropical crops (MAPA 2026; Junquera et al. 2024), is characterized by mild, wet winters, and hot, dry summers. The cold semi-arid climate (BSk) of La Almunia de Doña Godina features cold winters, and hot, dry summers with marked seasonal temperature contrasts and low annual precipitation (Peel et al. 2007).

Phenological observations of flower buds were carried out twice a week from bud break to flowering, considering full flowering (FP50) when 50% of flower buds were in stage F (Baggiolini 1952) that corresponds to stage 65 (full bloom) in the BBCH scale (Meier 2001; Fadón et al. 2015).

### Determination of endodormancy breaking

Flower bud growth was evaluated in response to warm conditions after field chilling exposure. Four shoots around 40 cm per cultivar were collected weekly from November to March, placed in a growth chamber (22 ± 1 °C, 12 h light photoperiod) and maintained on wet florist foam for 8 days. To determine the end of endodormancy, 10 flower buds were randomly picked and weighed on the first and last day in the growth chamber. Endodormancy was considered overcome when bud weight increased by more than 30% (Brown and Kotob 1957; Tabuenca 1964; Ruiz et al. 2007; Fadón and Rodrigo 2018; Guerrero et al. 2024). Using the endodormancy release date (T1) as reference, two additional time points were selected: two weeks before dormancy release was considered full dormancy (T0), and a bud weight increase exceeding 50% was used as the criterion for full recovery of activity (T2).

### Chilling and heat requirement estimation

Semi-hourly temperature data were recorded at local weather stations: station 31 (Épila) in Zaragoza (ORESA 2026), and a thermo-hygrometer placed at the experimental station of IHSM La Mayora in Málaga. For each cultivar and location, chill accumulation from September 1 to endodormancy release was quantified in chilling portions (CP) using the Dynamic Model (Fishman et al. 1987). Heat requirements were estimated in Growing Degree Hours (GDH) (Richardson et al. 1974), from endodormancy release to FP50.

### RNA extraction and library preparation

For each time point, flower buds were collected from multiple trees and pooled to create three replicates. Samples were flash frozen in liquid nitrogen and stored at − 80 ºC. Total RNA was isolated using the Total RNA Purification Kit (Norgen Biotek) and treated with TURBO DNA-free^TM^ kit (Ambion, Austin, TX, USA) to remove DNA contamination. Thirty-six samples containing 0.74–2.56 μg of RNA were submitted to Novogene (Cambridge, UK) for library preparation and RNA sequencing (Illumina NovaSeq 6000, 150 bp PE).

### mRNA-seq data processing

The *P. salicina* genome assembly and gene annotations were obtained from *P. salicina* Sanyueli Genome v2.0 (Liu et al. 2020), hosted in the Genome Database for Rosaceae (GDR; accession number tfGDR1044) (Jung et al. 2018). The quality of reads was assessed using FastQC v0.11.9 (Andrews 2010), and gene expression was quantified with RSEM v1.3.3 (Li and Dewey 2011) using STAR v2.7.9a as read aligner (Dobin et al. 2013). Gene counts in each dataset were normalized using the variance-stabilizing transformation (VST) method implemented in the DESeq2 v1.42.0 R package (Love et al. 2014), in the statistical software R (R Core Team 2023). Principal component analysis (PCA), hierarchical clustering, boxplots, heatmaps, and differential expression (DE) analysis were performed on VST-normalized counts.

### Co-expression network analysis

Gene co-expression network analysis was performed using the WGCNA (Weighted Gene Co-expression Network Analysis) R package v.1.74 (Langfelder and Horvath 2008). VST-normalized counts were used as input and analyzed separately for each climate condition. A soft-thresholding power was selected based on the scale-free topology criterion (*R*^2^ ≥ 0.7). Signed networks were constructed using Pearson correlation, and gene modules were identified by dynamic tree cutting, followed by merging at a height threshold of 0.25. Network construction was implemented using the blockwiseModules function, which computes the topological overlap matrix (TOM) in a block-wise manner.

Module eigengenes were correlated with two variables (cultivar and dormancy stage time point) using Pearson correlation, with cultivar encoded as a binary variable for this analysis. Modules showing the strongest positive and strongest negative significant (*p* < 0.005) associations with either variable were selected for downstream analysis. Hub genes were identified using two complementary metrics: genes were first ranked according to the combined measure of module membership and gene significance (|kME| ×|GS|), where GS was calculated relative to the trait most strongly associated with each module. In parallel, intramodular connectivity (kWithin) was derived from the adjacency matrix to assess network centrality. Network structure within selected modules was visualized based on adjacency-derived co-expression relationships using the igraph R package v.2.0.1.1 (Csárdi and Nepusz 2006), applying a fixed adjacency threshold of 0.2 for edge inclusion.

To facilitate biological interpretation, the top three genes per module, selected based on kWithin and |kME| ×|GS| rankings, were functionally annotated to assign gene identities and functional information to EMV.TU identifiers. This annotation was performed using Tripal MegaSearch, a data mining tool for bulk retrieval of genomic and functional information from Tripal-based databases (Jung et al. 2021).

### Differential expression analysis

Differential expression analyses were performed with DESeq2 v1.42.0 (Love et al. 2014). To gain a more detailed understanding of the regulatory mechanisms underlying dormancy, two types of analyses were conducted: differential expression analysis and functional enrichment analysis, both performed on the datasets from each location. First, to identify genes with dormancy stage-specific regulation within each location, gene expression at each time point was compared to the mean expression levels of the other two time points within each cultivar, allowing the detection of genes that define stage-specific transcriptional profiles. Furthermore, to assess the evolutionary dynamics of differentially expressed genes (DEGs) across stages, gene expression at time point T0 was compared with T1, and T1 was compared with T2. This pairwise design enables the identification of genes involved in stage-to-stage transitions, capturing dynamic regulatory changes that may be missed when analyzing each stage independently. Genes with a Benjamini–Hochberg (BH) adjusted *P-*value lower than 0.01 were selected as differentially expressed in the corresponding contrast.

To visualize the overlap among gene sets derived from the stage-specific differential expression analysis, UpSet plots were generated using the UpSetR package 1.4.0 in R (Conway et al. 2017). These plots were constructed based on binary presence/absence matrices derived from lists of significantly upregulated DEGs identified across dormancy stages in both cultivars under semi-arid and Mediterranean subtropical conditions. Specifically, each gene was assigned a value of 1 or 0 depending on whether it was classified as differentially expressed in a given contrast, allowing the identification of shared and condition-specific gene sets across the different analytical subsets.

### Phylogenetic analysis

Protein sequences of the two *DAM*-like genes identified in this study, together with the longest annotated homologous from related *Prunus* and other species retrieved from NCBI database (NCBI Resource Coordinators 2018), were aligned using CLUSTALX v.1.82 (Thompson et al. 1997). For nomenclature consistency, *DAM* genes previously described in *Prunus salicina* are referred to as *PsDAM*, whereas the *DAM*-like genes identified in this study are designated *PshDAM*, following the convention used in this work to distinguish previously published sequences from those newly identified in *P. salicina*. The alignments were subsequently edited as described previously (Lora et al. 2017) using GBLOCK v.0.91b (Castresana 2000; Talavera and Castresana 2007). Bayesian analysis was performed using MrBayes (Ronquist et al. 2012), following the JTT + Invariant (I) model of amino acid substitutions recommended by MEGA version X (Kumar et al. 2018). Trees were sampled every 100 generations for 1,000,000 generations in the Bayesian analysis, with the first 25% of the trees of each run discarded as burn-in.

### Functional analysis

Protein sequences used for functional analyses were derived from DEG sets identified in stage-specific contrasts. From these DEG sets, genes were further stratified according to the intersection structure obtained from the UpSet analysis. Although multiple intersection categories were generated, only selected groups corresponding to shared, location-specific, or cultivar-specific genes were retained for downstream analyses. Based on their expression profiles across dormancy progression, genes were subsequently categorized into dormancy stages.

The functional annotation of the obtained protein sequences was performed using the EggNOG database (evolutionary genealogy of genes: Non-supervised Orthologous Groups), version 5.0 (Huerta-Cepas et al. 2019). Protein sequences were analyzed with eggNOG-mapper (https://eggnog-mapper.embl.de/) with default parameters, adjusting the taxonomic scope per query to improve annotation accuracy, and transferring non-electronic Gene Ontology evidence to ensure higher-confidence functional assignments. Functional classification was further supported by the assignment of sequences to Clusters of Orthologous Groups (COG), which provide a hierarchical classification of proteins into broad functional categories based on orthology relationships. COG categories were used to summarize the distribution of predicted protein functions across the dataset. For this purpose, individual COG annotations were grouped into their respective functional classes, allowing comparative visualization of functional profiles among the analyzed sequences. Graphical representations of the functional distribution were generated using the ggplot2 package (Wickham 2016) in R (R Core Team 2023).

## Results

### Experimental establishment of endodormancy release

We first evaluated the phenology of two cultivars with different chilling requirements: "Hiromi Red" (HR; high chilling requirement) and "Crimson Glo" (CG; low-chilling requirement) under two locations in Spain with contrasting climates: La Almunia de Doña Godina, Zaragoza (semi-arid) and Algarrobo-Costa, Málaga (Mediterranean subtropical). Both cultivars are interspecific hybrids of *P. salicina* and other *Prunus* species, developed by Zaiger Genetics (Milošević and Milošević 2018).

In the semi-arid climate, full flowering (FP50) was observed in late February (February 24 for HR; February 19 for CG). In the Mediterranean subtropical climate, flowering was delayed: FP50 occurred 20 days later in HR (March 16) and 11 days later in CG (March 02).

**Fig. 1:**
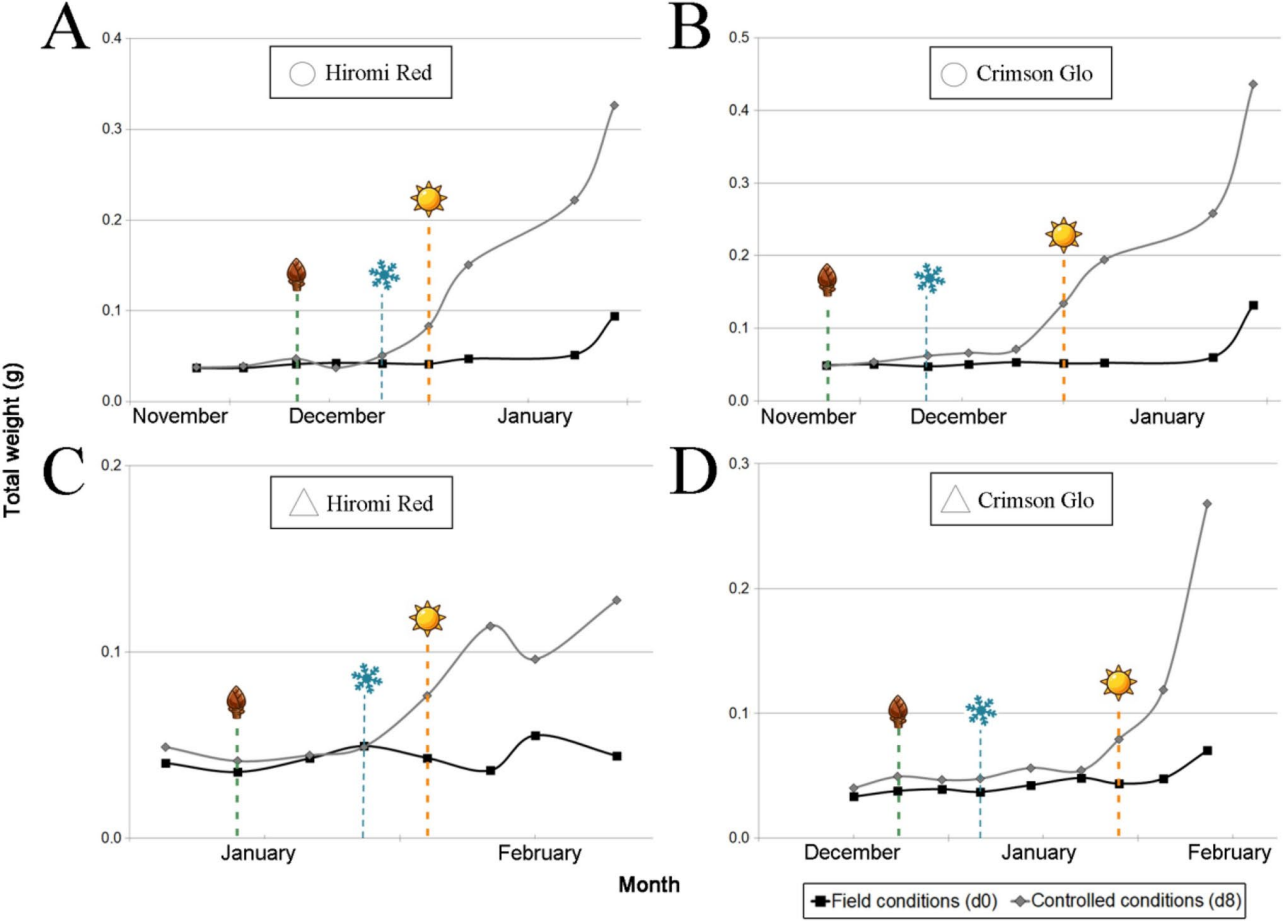
Estimation of breaking of endodormancy date for two Japanese plum cultivars ("Hiromi Red" and "Crimson Glo") under semi-arid (○) and Mediterranean subtropical (△) climates using an experimental methodology. The icons represent the dates of dormancy stages: full dormancy (T0, ), endodormancy release (T1, ), and full recovery of activity (T2, ). The lines represent the flower bud weight under field conditions (black square) and after 8 days in a growth chamber (gray rhombus). Chilling requirements were considered fulfilled when the increase in flower bud weight in the growth chamber was 30% greater than that observed under field conditions (*n* = 10). **A** Hiromi Red under semi-arid climate. **B** Crimson Glo under semi-arid climate. **C** Hiromi Red under Mediterranean subtropical climate. **D** Crimson Glo under Mediterranean subtropical climate

In the semi-arid climate, HR and CG reached endodormancy release on December 23 and 10, respectively. Full dormancy dates were set on December 10 for HR and November 25 for CG. Full recovery of bud activity was recorded on December 30 in both cultivars. However, in HR this recovery occurred one week after dormancy release, while in CG it took three weeks (Fig. 1A, B). A similar but delayed trend was observed in the Mediterranean subtropical climate, where all three time points occurred approximately one month later than in the semi-arid climate. The delay was 35 days for HR and 26 days for CG (Fig. 1C, D).

Chilling and heat requirements varied across cultivars and climates. Under semi-arid conditions, CG required 32 chilling portions (CP) and HR 42 CP, whereas under Mediterranean subtropical conditions, CP values were considerably lower (15 CP for CG, 26 CP for HR). In contrast, heat requirement values were up to 60% higher in the Mediterranean subtropical climate: CG 11,395 vs. 6998 GDH, and HR 10,878 vs. 6,567 GDH (Mediterranean vs. semi-arid).

### Transcriptomic overview of flower bud development during dormancy

The number of mRNA-seq aligned reads to the genome per sample ranged from 41.7 M to 57 M, with alignment rates between 84.4% and 85.8% (Fig. S2, Table S1), suggesting that the reference genome adequately represents the transcriptomic data. Boxplot of VST-normalized counts revealed similar expression distributions across all samples and replicates, with median expression values closely aligned across conditions, indicating comparable overall gene expression levels. However, differences were observed in the number and dispersion of outliers (Fig. S3A). The three replicates clustered closely together, as shown by both the hierarchical clustering dendrogram (Fig. S3B) and the principal component analysis (PCA) plot (Fig. S3C). The first principal component (PC1, 38%) clearly separated the two cultivars while the second principal component (PC2, 29.8%) showed a separation based on climate, regardless of the dormancy stage. Interestingly, CG at full dormancy and HR at endodormancy release under Mediterranean subtropical conditions clustered more closely with semi-arid climate samples than with other samples within the same climate under different dormancy stages. In addition, the heatmap (Fig. S3D) also showed high consistency among replicates, and the gene expression patterns supported the PCA results, suggesting specific transcriptomic responses related to genotype and environment during dormancy.

### Construction of co‑expression modules and functional analysis

Beyond sample-level transcriptomic structure, we next investigated coordinated gene regulation using a co-expression network analysis. WGCNA identified independent co-expression networks for semi-arid and Mediterranean subtropical climate conditions. The resulting networks satisfied the scale-free topology criterion, with soft-thresholding powers of 3 and 7 for the semi-arid and Mediterranean subtropical datasets, respectively (Figs. S4A, S5A). Network construction identified 12 co-expression modules in the semi-arid and 30 in the Mediterranean subtropical climate conditions, as shown in the module size distributions (Figs. S4B, S5B).

**Fig. 2:**
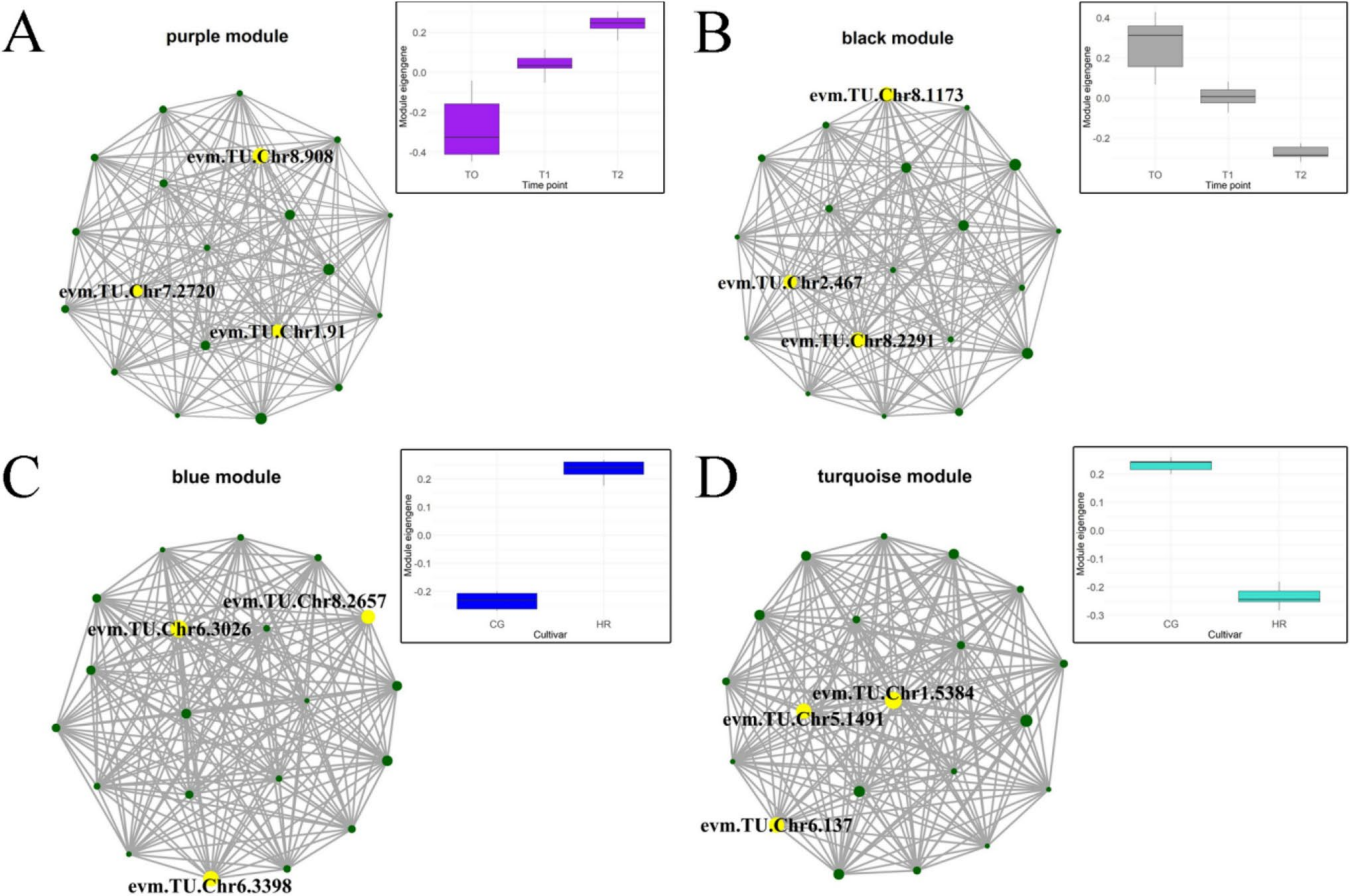
Visualization of selected co-expression modules and hub genes under semi-arid climate conditions. **A**–**D** Top 20 genes ranked by intramodular connectivity in modules correlated with dormancy stage (purple, positive; black, negative) or cultivar (blue, positive; turquoise, negative). The characteristic expression profile of each module relative to the associated variable is shown. Hub genes (top 3) are highlighted in yellow, and node size is proportional to intramodular connectivity. The annotated hub genes are purple module (*evm.TU.Chr1.91, evm.TU.Chr7.2720, evm.TU.Chr8.908*), black module (*evm.TU.Chr2.467, evm.TU.Chr8.1173, evm.TU.Chr8.2291*), blue module (*evm.TU.Chr6.3026, evm.TU.Chr6.3398, evm.TU.Chr8.2657*), and turquoise module (*evm.TU.Chr1.5384, evm.TU.Chr5.1491, evm.TU.Chr6.137*)

Module–trait association analysis revealed structured relationships between co-expression modules and the studied variables (Figs. S4C, S5C). Module eigengene expression profiles showed comparable temporal patterns across both climates (Figs. 2, S6). In each condition, modules positively correlated with dormancy stage increased progressively over time (Figs. 2A, S6A), whereas negatively correlated modules showed the opposite trend (Figs. 2B, S6B), indicating similar transcriptional dynamics at the network level. However, differences in the distribution of eigengene values across stages were observed: in the semi-arid conditions, higher eigengene values were detected at T0 (Figs. 2A, B), whereas in the Mediterranean subtropical conditions, higher values were observed at later stages (T1–T2) (Figs. S6A, B). Additionally, cultivar-specific differences were consistent across climates, with the low-chilling-requirement cultivar (CG) showing lower eigengene values in positively correlated modules and higher values in negatively correlated ones compared to the high-chilling-requirement cultivar (HR), indicating differences in the magnitude of transcriptional responses (Figs. 2C, D and S6C, D).

The complete gene lists from the selected modules were extracted for downstream network and functional analyses (Tables S2–5). Hub gene prioritization identified hub genes for each variable-associated module based on their network connectivity and module–trait association.

In the semi-arid climate, modules correlated positively (purple) and negatively (black) with dormancy stage showed distinct functional enrichment. GS×kME and kWithin consistently highlighted key hubs such as *Protein Arginine Methyltransferase 10* (*PRMT10*) and glycosyl hydrolases, which are involved in signaling, carbohydrate metabolism, cell wall dynamics, and protein turnover (Table S6, Fig. 2A, B). Cultivar-associated modules also showed strong agreement between correlation structure and hub ranking: positively correlated modules (blue) highlighted *Ubiquitin-Specific Protease 13* (*USP13*) and *WD40* repeat proteins linked to RNA processing and ubiquitin regulation (Table S6, Fig. 2C), while negatively correlated modules (turquoise) included stress- and carbohydrate-related genes supported by high GS×kME and connectivity (Table S6, Fig. 2D).

In the Mediterranean subtropical climate, modules positively correlated with dormancy stage (blue) identified metabolic hub genes such as *Rubisco activase*, *histidine kinase 3*, and *D-glycerate 3-kinase*, associated with photosynthesis, carbon metabolism, and signaling (Table S6; Fig. S6A). Negatively correlated modules (turquoise) highlighted hub genes like *monodehydroascorbate reductase 4*, *carotenoid cleavage dioxygenase 1*, and *protodermal factor 1*, linked to redox balance, hormone regulation, and development (Table S6, Fig. S6B). Cultivar-associated modules again showed strong concordance between correlation and GS×kME ranking: positively correlated modules (green) included *USP13*, *MBOAT*, and glycosyl hydrolases involved in proteostasis, lipid remodeling, and cell wall processes (Table S6; Fig. S6C), while negatively correlated modules (yellow) contained bZIP transcription factor networks and membrane lipid–associated genes (Table S6; Fig. S6D).

The cultivar-associated GS×kME hub evm.TU.Chr7.2928 (a glycosyl hydrolase with a chitinase domain) was shared across both climates, suggesting a conserved role in cell wall remodeling and stress-related carbohydrate metabolism across environments. Several GS×kME-selected genes are also key network hubs, showing strong connectivity and repeated presence across modules and functions in both climates. These included regulatory and structural proteins such as *WD40* repeat proteins (evm.TU.Chr6.3026), small acidic proteins (evm.TU.Chr1.5384), and metabolic regulators like *MBOAT* (evm.TU.Chr5.1545), as well as conserved hubs shared in semi-arid and Mediterranean subtropical conditions such as glycosyl hydrolases, nucleotide-diphospho-sugar transferases, and *USP13* (Table S6).

### Transcriptomic insights into dormancy regulation in the semi-arid climate

**Fig. 3:**
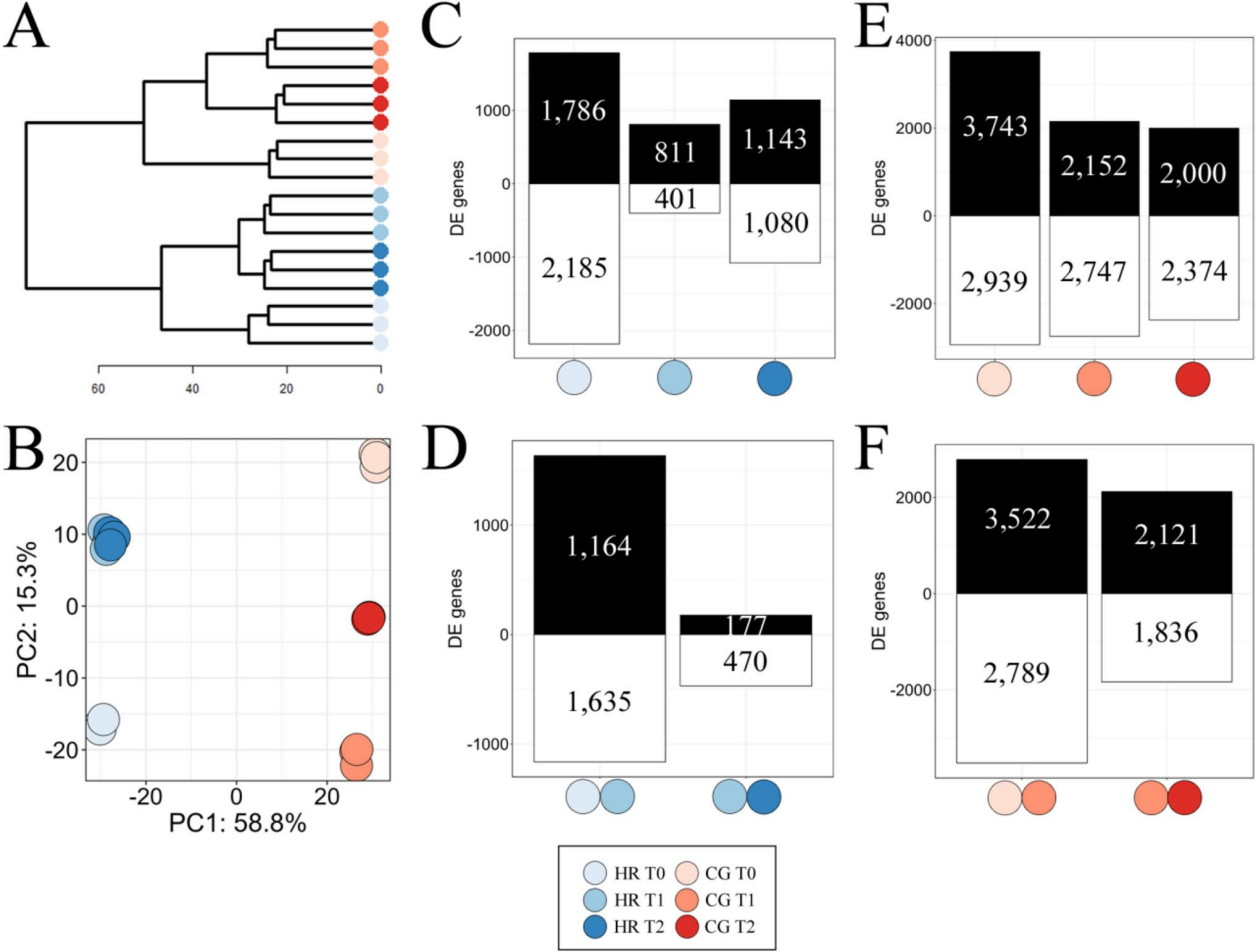
mRNA-seq dataset overview for Japanese plum cultivars "Hiromi Red" (blue) and "Crimson Glo" (red) under semi-arid climate conditions across three dormancy stages. **A** Hierarchical clustering dendrogram and **B** PCA of the mRNA-seq libraries, both showing that replicates are similar with each other. **C** Number of significant stage-specific genes in "Hiromi Red" (adjusted *P*-value < 0.01; black: upregulated genes; white: downregulated genes). See Table S7 for the gene list. **D** Number of DEGs between early and mid-stages, and between mid- and late stages in "Hiromi Red" (adjusted *P*-value < 0.01; black: upregulated genes; white: downregulated genes). See Table S8 for the gene list. **E** Number of significantly stage-specific genes in "Crimson Glo" (adjusted *P*-value < 0.01; black: upregulated genes; white: downregulated genes). See Table S9 for the gene list. **F** Number of DEGs between early and mid-stages, and between mid- and late stages in "Crimson Glo" (adjusted *P*-value < 0.01; black: upregulated genes; white: downregulated genes). See Table S10 for the gene list. Full dormancy (T0): light color; dormancy release (T1): normal color, and full recovery (T2): darker color

In the semi-arid location, clustering analyses and PCA showed clear differentiation between cultivars and dormancy stages (Fig. 3). In CG, T1 and T2 were similar, indicating a higher transcriptomic shift from T0 to T1. Both the dendrogram (Fig. 3A) and the first principal component (Fig. 3B) showed a clear differentiation between the two cultivars. However, the second principal component showed a clear differentiation among the three dormancy stages in the low-chill cultivar (CG), whereas in the high-chill cultivar (HR), the T1 and T2 stages did not separate clearly.

Analysis of HR samples showed fewer DEGs across dormancy stages than CG. The highest number of DEGs in HR occurred during full dormancy (3971 genes, T0) and after dormancy release (2223 genes, T2) compared to the dormancy release stage (1212 genes, T1) (Fig. 3C, Table S7). There was a marked difference between periods, with a 77% reduction of DEGs after dormancy release (647 genes) compared to the period before dormancy release (2799 genes) (Fig. 3D, Table S8).

In contrast, CG exhibited a higher number of DEGs across all stages, with the largest number at full dormancy (6682 genes, T0) (Fig. 3E, Table S9). Compared to HR, the number of DEGs, including both up- and downregulated genes, was more than double before dormancy release (6311 genes) and after dormancy release (3957 genes) (Fig. 3F, Table S10).

Functional enrichment analysis based on Cluster of Orthologous Groups (COG) categories showed a progressive decrease in the number of DEGs across most categories during dormancy in HR, followed by an increase upon the reactivation of activity (Fig. S7A, B, Table S11). At full dormancy (T0), a large number of genes were downregulated, particularly those involved in transcription, signaling, and protein regulation (Fig. S7A, Table S11).

In contrast, CG exhibited a progressive increase in the number of downregulated genes across dormancy stages, mainly in categories related to cellular processes, signaling, and metabolism, while genes involved in information storage and processing remained active initially but were progressively downregulated as dormancy progressed, (Fig. S8A, Table S12). At full dormancy, a higher number of upregulated genes were observed, particularly in categories related to signaling, protein regulation, transcription, and metabolism, although their expression gradually decreased over time. Conversely, genes related to cell cycle reactivation and preparation for active growth—such as those involved in translation, replication, and chromatin modification—became increasingly expressed, with their activity sustained until T2 (Fig. S8B, Table S12).

### Transcriptomic insights into dormancy regulation in the Mediterranean subtropical climate

**Fig. 4:**
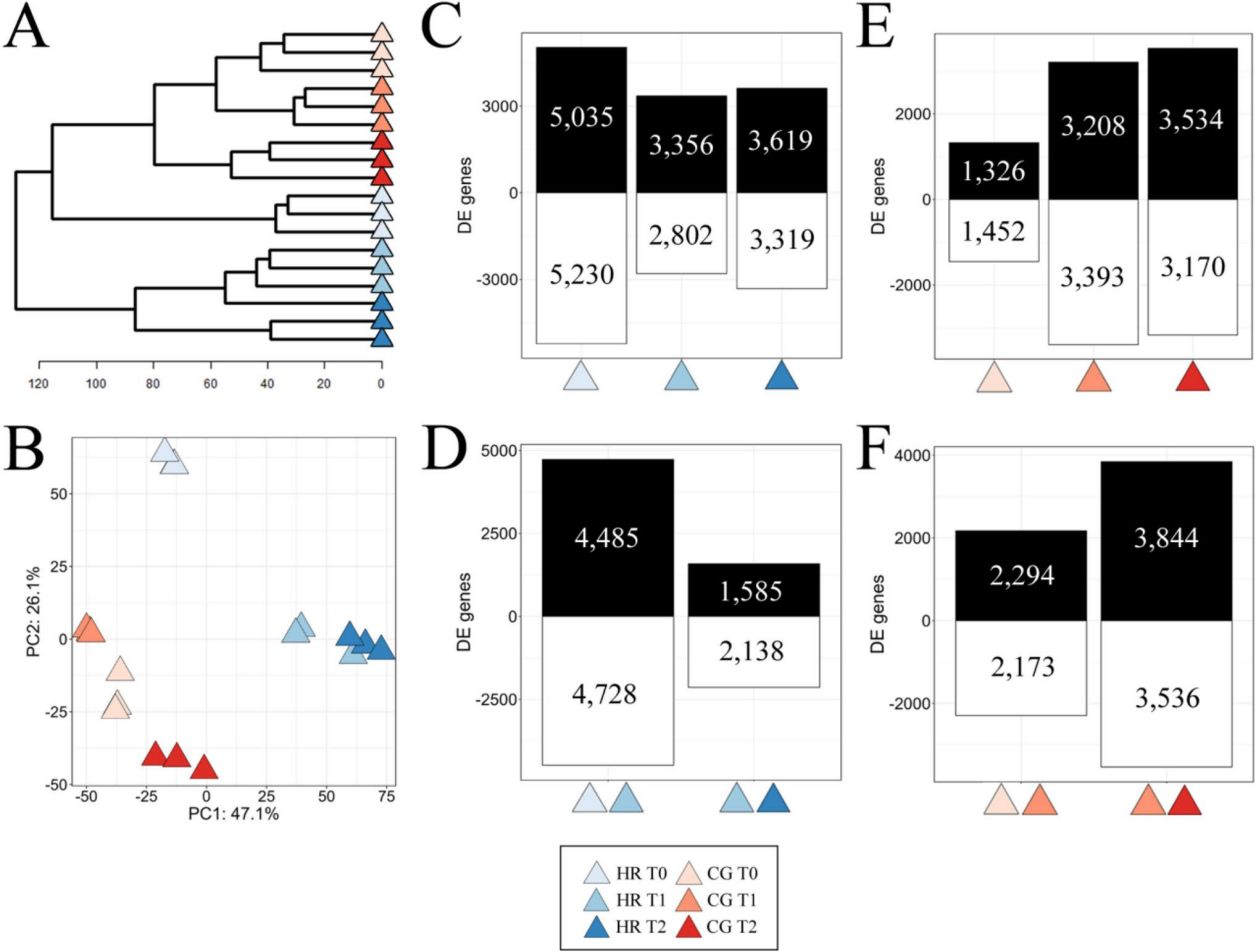
mRNA-seq dataset overview for Japanese plum cultivars "Hiromi Red" (blue) and "Crimson Glo" (red) under Mediterranean subtropical climate conditions across three dormancy stages. **A** Hierarchical clustering dendrogram and **B** PCA of the mRNA-seq libraries, both showing that replicates are similar with each other. **C** Number of significant stage-specific genes in "Hiromi Red" (adjusted *P*-value < 0.01; black: upregulated genes; white: downregulated genes). See Table S13 for the gene list. **D** Number of DEGs between early and mid-stages, and between mid- and late stages in "Hiromi Red" (adjusted *P*-value < 0.01; black: upregulated genes; white: downregulated genes). See Table S14 for the gene list. **E** Number of significantly stage-specific genes in "Crimson Glo" (adjusted *P*-value < 0.01; black: upregulated genes; white: downregulated genes). See Table S15 for the gene list. **F** Number of DEGs between early and mid-stages, and between mid- and late stages in "Crimson Glo" (adjusted *P*-value < 0.01; black: upregulated genes; white: downregulated genes). See Table S16 for the gene list. Full dormancy (T0): light color; dormancy release (T1): normal color, and full recovery (T2): darker color

In the Mediterranean subtropical climate, a similar gene expression pattern to that observed in the semi-arid climate was detected, with a clear separation between the two cultivars (PC1, 47.1%), as well as a distribution based on the dormancy stage (PC2, 26.1%) (Fig. 4A, B). However, the samples corresponding to full dormancy in HR clustered closer to CG. A significant difference in the number of DEGs was observed between the two cultivars during the dormancy stage (T0). In HR, 5035 genes showed significantly increased expression, while 5230 genes showed significantly decreased expression. In contrast, CG exhibited a much smaller number of DEGs, with 1326 genes upregulated and 1452 downregulated. The number of DEGs was similar between the two cultivars for T1 and T2 (Fig. 4C–F, Tables S13–16).

In HR, COG category analysis revealed a progressive decrease in the number of DEGs from T0 to T2 (Fig. S9A, B). At full dormancy (T0), a large number of genes were downregulated, particularly in categories related to transcription, signal transduction mechanisms, and posttranslational modification, protein turnover, and chaperones (Fig. S9A, Table S17). In parallel, the number of upregulated genes gradually increased from T0 onward, especially in categories involved in metabolism and transcription (Fig. S9B, Table S17).

In CG, COG annotation revealed a distinct gene regulation pattern compared to HR, suggesting a progressive general reactivation that intensified once dormancy was overcome. At full dormancy (T0), although several functional categories such as signaling and protein regulation, transcription, and carbohydrate metabolism were downregulated (Fig. S10A, Table S18), these same categories also showed a notable number of upregulated genes (Fig. S10B, Table S18). As CG progressed to T1 and T2, the number of upregulated DEGs increased across multiple COG categories.

### Differential expression of key dormancy-related genes

In addition to the global transcriptomic analyses, which provided an overall view of gene expression dynamics during dormancy transitions, we specifically examined the expression patterns of well-known dormancy-related genes to better understand these expression changes. These candidate genes were selected based on their previously reported roles in dormancy regulation and their relevance within the DEG sets identified in our analysis. Among them, we analyzed genes such as *STRESS-ASSOCIATED PROTEIN1* (*SAP1*) (Prupe.2G010400) (Lloret et al. 2017), *ALLENE OXYDE CYCLASE* (*AOC*)-*like 1–2* (Prupe.1G306100, Prupe.3G239900) (Lloret et al. 2021), *DAM* (Bielenberg et al. 2004, 2008), *FLOWERING LOCUS T* (*FT*) (Prupe.6G364900) (Hsu et al. 2011; Hao et al. 2015), *TARGET OF RAPAMYCIN* (*TOR*)-*like* (Prupe.8G151300) (Menand et al. 2002; Lloret et al. 2017) *TONOPLAST INTRINSIC PROTEIN* (*TIP*)-*like* (Prupe.2G229500) (Ludevid et al. 1992; Lloret et al. 2017), and *LIPOXYGENASE* (*LOX*)-*like* (Prupe.2G005300) (Lloret et al. 2021). To this end, we first searched for the putative orthologs of these genes in our transcriptome using their sequences from *Prunus persica*. For most candidate genes, a single sequence was identified, except for the *DAM* genes, for which two *DAM-like* sequences were found. Phylogenetic analysis placed one of the two putative *DAM* ortholog (*evm.model.Chr1.599_syl_v2.0*) within the *DAM6* clade, and it was subsequently renamed as *PshDAM6*. Similarly, the other putative ortholog of *DAM* (*evm.model.Chr1.600.601.603.604_syl_v2.0*) was placed within the *DAM1* clade and renamed as *PshDAM1* (Fig. S11).

**Fig. 5:**
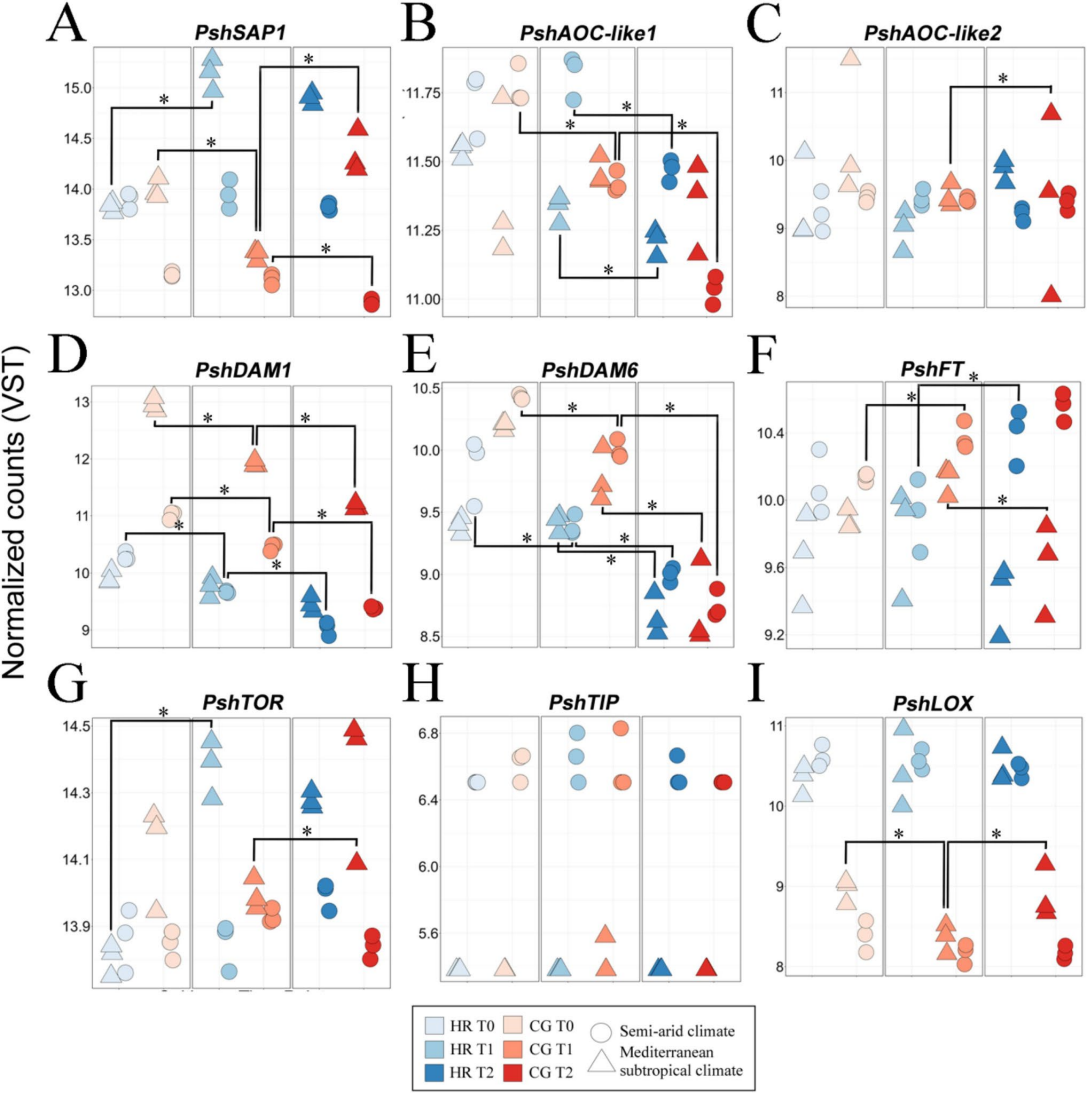
Normalized expression counts by VST for Japanese plum cultivars "Hiromi Red" (blue) and "Crimson Glo" (red) under semi-arid and Mediterranean subtropical climate conditions across three dormancy stages in known markers: **A** *STRESS-ASSOCIATED PROTEIN1* (*SAP1*); **B** *ALLENE OXIDE CYCLASE* (*AOC*)-like 1; **C** *ALLENE OXIDE CYCLASE* (*AOC*)-like 2; **D** *PshDAM1*; **E** *PshDAM6*; **F** *FLOWERING LOCUS T* (*FT*); **G** *TARGET OF RAPAMYCIN* (*TOR*)-like; **H** *TONOPLAST INTRINSIC PROTEIN* (*TIP*)-like; and **I** *LIPOXYGENASE* (*LOX*)-like. Full dormancy (T0): light color; dormancy release (T1): normal color, and full recovery (T2): darker color. Semi-arid climate is represented by circles (○), while Mediterranean subtropical climate is represented by triangles (△)

Significant differences in expression were observed in all key dormancy-related genes, except *TIP* (Fig. 5). *PshDAM1* expression decreased across dormancy stages, more pronounced in CG and in the Mediterranean subtropical climate. *PshDAM6* followed a similar pattern, showing reduced expression at T2, with a steeper decrease in CG and a similar trend under both climates.

*FT* expression increased at T2 under semi-arid conditions but decreased at T2 in the Mediterranean subtropical climate. *SAP1* was upregulated in HR during dormancy release in Mediterranean subtropical conditions but fluctuated in CG. In the semi-arid climate, *SAP1* expression levels were generally lower, with significant differences observed only after dormancy release in CG. No significant differences were found in *TIP* expression across stages, cultivars, and climates. *TOR* expression increased after dormancy release, particularly in the Mediterranean subtropical climate. *LOX* expression was similar across all dormancy stages in both cultivars, regardless of location, with higher expression levels in HR. However, significant differences were only observed for CG in the Mediterranean subtropical climate. In *AOC-like1*, a reduction in expression was observed across dormancy stages in both climates, with significant differences at dormancy release. For *AOC-like2*, expression levels remained similar across stages in both climates, with significant differences only observed at the resumption of growth in CG under Mediterranean subtropical conditions.

### Cultivar- and climate-specific upregulated gene expression

**Fig. 6:**
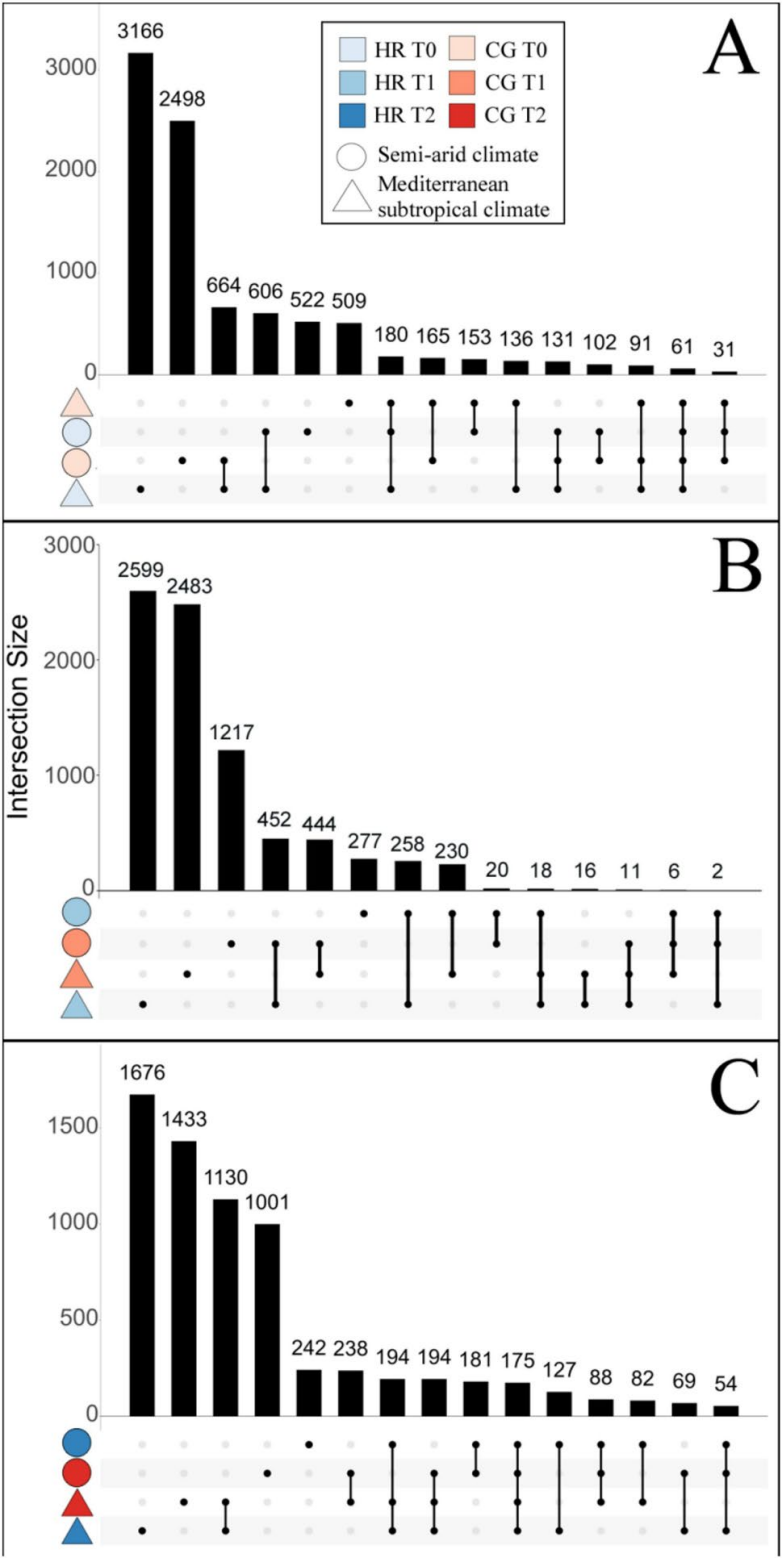
UpSet plots showing the DEG profiles across three dormancy stages: **A** Full dormancy, **B** Dormancy release, and **C** Full recovery. Each plot shows the comparison of the DEGs in both Japanese plum cultivars "Hiromi Red" and "Crimson Glo" under both climate conditions; semi-arid (○) and Mediterranean subtropical (△). The intersections in the UpSet plots highlight the overlap and unique DEG sets across cultivars and climate conditions at each stage, with the bars representing the number of DEGs in each combination

To explore how gene expression is modulated in response to climatic conditions and genetic background, we analyzed stage-specific sets of upregulated DEGs across dormancy stages in both cultivars under semi-arid and Mediterranean subtropical environments. During dormancy (T0), HR showed 3,166 DEGs upregulated under Mediterranean subtropical conditions, whereas only 522 were observed under semi-arid conditions. Interestingly, 606 genes were shared between the two climates. In contrast, in CG, 509 DEGs upregulated were found under Mediterranean subtropical conditions, and 2498 under semi-arid conditions, with an overlap of only 165 genes between the two climates. Additionally, 136 DEGs were identified between the two cultivars in the Mediterranean subtropical climate, and 102 DEGs in the semi-arid climate. Across both cultivars, 61 DEGs were common to both climates (Fig. 6A, Tables S19–23). This contrasting pattern indicates that dormancy maintenance relies on cultivar-specific transcriptional strategies that are differentially modulated by climate.

During dormancy release (T1), no common upregulated DEGs were observed between cultivars and climates. Few DEGs were identified within each climate: 20 in the semi-arid climate and 16 in Mediterranean subtropical climate. However, the number of upregulated DEGs for each cultivar, regardless of climate, was high: 444 for CG and 258 for HR (Fig. 6B, Tables S24–27). This result suggests that the transition from dormancy to dormancy release is predominantly controlled by genetic background, with minimal influence of climatic conditions.

After dormancy release (T2), more upregulated DEGs were associated with Mediterranean subtropical conditions. Overall, CG exhibited twice as many DEGs as HR (Fig. 6C, Tables S28–32). This pattern indicates that climate-dependent transcriptional regulation becomes more relevant once dormancy has been overcome, particularly during the resumption of active growth.

Altogether, the subdivision of DEGs in Fig. 6 reveals stage-specific shifts in the relative contribution of genotype and climate to transcriptional regulation across dormancy transitions.

**Fig. 7:**
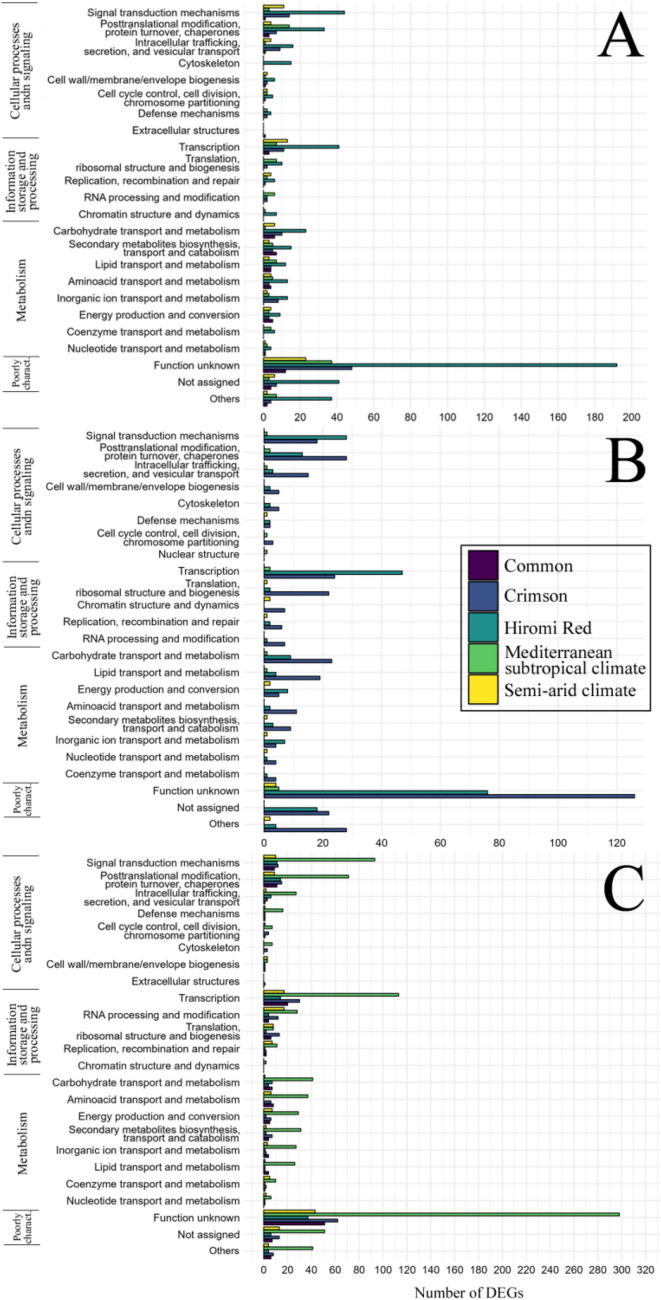
Number distribution of Clusters of Orthologous Groups (*COG*) functional classification analysis among 23 categories using eggNOG-mapper. **A** Full dormancy, **B** Dormancy release, and **C** Full recovery

To further evaluate gene function across various categories from the COG database, the majority of genes were grouped into three major functional categories: metabolism (13–49%), information storage and processing (5–30%), and cellular processes and signaling (6–40%) (Fig. 7, Tables S33–35). Genes with unclassified functions were assigned to poorly characterized categories, while those mixed or overlapping roles were grouped under "Others" (52 categories, 2–13%). Overall, genes were distributed across twenty-three specific COG functional classes. Across all conditions, a substantial proportion had unknown (22–35%) or unassigned functions (2–8%), indicating that several biological processes remain poorly understood. Functional category distributions were generally consistent, except during dormancy, when metabolic genes dominated (49%).

The COG functional classification analysis revealed that most genes were associated with the categories "Carbohydrate transport and metabolism," "Transcription," "Signal transduction mechanisms," and "Posttranslational modification, protein turnover, chaperones." Notable inactivity was observed in categories related to defense mechanisms and secondary metabolite biosynthesis, suggesting a lack of active stress response during this state. Furthermore, low values in categories related to cell division, cytoskeleton organization, and extracellular structures reflected limited growth and expansion over dormancy stages (Fig. 7, Tables S33–35).

## Discussion

We analyzed the transcriptomic profiles of two Japanese plum cultivars, "Hiromi Red" and "Crimson Glo," which have high and low chilling requirements, respectively, under semi-arid and Mediterranean subtropical climates. Our findings revealed that climate significantly impacts dormancy progression and gene expression patterns in these cultivars, with transitions between the three dormancy phases occurring approximately one month later under Mediterranean subtropical conditions. These patterns are consistent with studies in temperate woody species showing that insufficient winter chilling can delay, rather than advance, spring phenology, with later budburst and higher heat requirements in low-chill or subtropical environments (Wang et al. 2020; Xu et al. 2021). Discrepancies were observed in the quantification of chilling requirements, with a remarkable reduction in the CP required in the Mediterranean subtropical climate for both cultivars, while their CPs in the semi-arid climate aligned with previous studies (Guerrero et al. 2024).

### Dormancy dynamics and transcriptomic analysis across climates

Traditionally considered as a period of inactivity, dormancy is now recognized as a dynamic and complex process involving extensive cellular and molecular activities (Rohde and Bhalerao 2007; Zhao et al. 2025a). Transcriptomic studies in *Prunus* have revealed that both endodormant and ecodormant flower buds exhibit unique and fluctuating transcriptional profiles, with continuous metabolic activities such as starch accumulation (Fadón et al. 2018; Yu et al. 2020; Calle et al. 2022), hormonal fluctuations (Vimont et al. 2019; Canton et al. 2021; Liu et al. 2025), and floral structure differentiation (Lloret et al. 2018; Zhao et al. 2025b). Consistently, our co-expression network analysis showed that dormancy progression across climates is governed by coordinated gene modules rather than isolated responses. Module eigengene profiles revealed similar temporal organization patterns in both environments, indicating coordinated activation and repression processes during dormancy progression. Despite these similarities, differences in eigengene distribution across stages suggest that environmental conditions modulate the dynamics of these transcriptional programs. In addition, cultivar-specific patterns were maintained across climates, with the high chilling requirement cultivar showing stronger activation of positively correlated modules, while the low-chilling-requirement cultivar displayed the opposite trend. Together, these results indicate that climate-specific differences in network structure are associated with processes such as carbohydrate metabolism, signaling, redox processes, and protein turnover, while also suggesting the presence of common regulatory strategies across environments. Overall, these findings support the view that transcriptional regulation during dormancy operates at the level of coordinated gene networks rather than depending on specific individual genes.

Previous transcriptomic studies in other *Prunus* species have identified hundreds to thousands of DEGs at different dormancy stages (Zhu et al. 2015; Yu et al. 2020) while fewer than 100 DEGs were expressed during full endodormancy. A meta-analysis of RNA-Seq studies in *Prunus* species (including apricot, almond, peach, and sweet cherry) identified 6,860 genes related to endodormancy, revealing conserved pathways involved in cold acclimation, cell growth control, oxidative signals, soluble sugar regulation, and phytohormone signaling, suggesting that common molecular mechanisms underlie dormancy regulation within this genus. However, the number of DEGs varies among species, cultivars, and dormancy stages (Calle et al. 2022).

In our study, the high-chill cultivar, "Hiromi Red," exhibited a more pronounced reduction in DEGs between the dormancy phases, with a notably higher number of DEGs in both climates compared to previous research in Japanese plum (Fang et al. 2022). For the low-chill cultivar, "Crimson Glo," the pattern of DEGs in the semi-arid climate was consistent with that observed for "Sanyueli" (Fang et al. 2022), showing a gradual decrease in DEGs during the transition between endodormancy and dormancy release. However, in the Mediterranean subtropical climate, an increase in DEGs was observed after dormancy release.

### Transcriptomic differentiation influenced by climate and dormancy stage

Despite recent RNA-seq studies focusing on dormancy control in *Prunus* species, our study addresses a significant gap by analyzing transcriptomic responses across different climates using two cultivars with different chilling requirements. PCA results revealed that climate had a greater influence on transcriptomic differentiation than dormancy stage, suggesting that the environmental conditions have a more significant impact on the transcriptomic profiles than the progression of dormancy. This contrasts with previous studies in other *Prunus* species, such as apricot and peach, where developmental stages had a greater influence on the transcriptomic profile (Yu et al. 2020).

Among the DEGs, several key regulators of dormancy were selected as representative of the main biological processes associated with dormancy transitions and climatic conditions. For example, Lloret et al. (2017) showed that *PpSAP1* in peach is expressed in dormant buds and expression decreases upon dormancy release. In our study, both cultivars showed a similar decreasing expression trend of *PpSAP1*, but only under semi-arid conditions. Conversely, under Mediterranean subtropical conditions, a significant increase in *PshSAP1* expression was observed, particularly in CG before dormancy release and in HR after dormancy release, suggesting an adaptive role in the adaptation of Japanese plum to different climatic conditions, especially in warmer winter environments, consistent with the described involvement of *PpSAP1* in stress tolerance and cell growth (Lloret et al. 2018). In contrast to previous reports in European plum and peach, where higher expression levels of *PshTIP* were detected after dormancy release, we found no significant differences in *PshTIP* expression, (Lloret et al. 2017; Quesada-Traver et al. 2020). However, an increase in *PshTOR* expression was observed in both cultivars in the Mediterranean subtropical climate, following a pattern similar to the expression of *PshSAP1*, supporting the idea that climate-associated signaling and growth-regulatory pathways are differentially engaged across environments.

The *AOC-like 1–2* and *LOX-like* genes, which are involved in jasmonic acid biosynthesis (Berni et al. 2019), have also been linked to dormancy and related physiological processes in other *Prunus* species (Lloret et al. 2021). Prudencio et al. (2021) observed a decrease in *LOX3.1* expression during dormancy release in almond, suggesting that lipoxygenase-mediated lipid peroxidation provides fatty acids for energy production during this process. In peach, an increase in *LOX-like, AOC-like1*, and *AOC-like2* expression has been reported during dormancy release, with a positive correlation with *PpeDAM6* expression, suggesting these genes may play a role in dormancy release (Lloret et al. 2021; Puertes et al. 2023). In our study, we observed minimal expression changes across dormancy stages for these genes. However, *PshAOC-like1* followed a decreasing trend similar to that reported for *LOX3.1* in almond (Prudencio et al. 2021), indicating that similar mechanisms might be at play in dormancy release across different *Prunus* species.

However, it is important to highlight that the expression patterns identified in this study are based on RNA-seq data, which provide a robust transcriptomic framework for candidate gene selection. These results establish a solid basis for future work, where targeted functional characterization will allow a more detailed understanding of the specific roles of these genes in dormancy regulation.

### *FT* and *DAM* genes: expression patterns and phylogenetic insights

*FT-like*, a key regulator of growth and flowering processes (Fadón et al. 2020a), showed increased expression after dormancy release under semi-arid conditions, consistent with previous reports in sweet cherry (Canton et al. 2021). However, we observed a decrease in *FT* expression after dormancy release in the Mediterranean subtropical climate. On the other hand, the *DAM* genes, which have been identified as key regulators of the dormancy cycle and climate adaptation (Goeckeritz and Hollender 2021), were downregulated in our study after prolonged exposure to cold temperatures, an essential factor for triggering dormancy release in Rosaceae (Falavigna et al. 2019). These findings align with those reported in other fruit tree species, where *DAM* gene expression correlates with the downregulation of *FT* during dormancy (Lloret et al. 2018; Puertes et al. 2023). In both semi-arid and Mediterranean subtropical climates, *DAM* genes showed high expression in dormant buds and low expression after the fulfillment of chilling requirements, which is consistent with previous reports in different species of the Rosaceae, such as Japanese plum (Fang et al. 2022), European plum (Quesada-Traver et al. 2020), sweet cherry (Rothkegel et al. 2017; Vimont et al. 2019), peach (Jiménez et al. 2010), Japanese apricot (Zhang et al. 2018), and apple (Mimida et al. 2015). Interestingly, while a significantly lower *DAM* expression was reported in the low-chill Japanese plum cultivar "Sanyueli" (Fang et al. 2022), our results showed lower *DAM* expression in the high-chill cultivar, Hiromi Red. These differences may be influenced by epigenetic factors that modulate *DAM* gene expression (Ríos et al. 2014), in response to the specific environmental conditions of each location.

Our phylogenetic analysis revealed that DAM proteins in the Amygdaloideae subfamily of Rosaceae originated from the SHORT VEGETATIVE PHASE (SVP) 2 clade, which also includes AGAMOUS-LIKE (AthAGL24) of *Arabidopsis thaliana* (Quesada-Traver et al. 2022). In several *Prunus* species six *DAM* genes have been identified, contrasting with the lower number of *DAM* genes found in annual model plants (Fang et al. 2022; Quesada-Traver et al. 2022). The expansion of the *DAM* genes may have resulted from serial tandem duplications occurring before the diversification of the *Prunus* genus (Jiménez et al. 2009). In our transcriptomic data, we surprisingly detected only *PshDAM1* and *PshDAM6*. However, in the currently available *Prunus salicina* ‘Sanyueli’ genome, *DAM* homologs are represented by a single predicted gene model, suggesting that the limited detection of *DAM* genes likely reflects constraints of the current genome assembly and annotation rather than the true *DAM* gene complement. Additional *DAM* genes may be resolved once improved genome assemblies and more curated annotations become available.

Interestingly, the phylogenetic analysis of *DAM* genes revealed that *PsDAM* genes from the Japanese plum cultivar "Sanyueli" clustered in a separate clade from the *DAM* sequences, including *PshDAM1* and *PshDAM6*. Moreover, *PsDAM6* exhibited insertions in its introns and a deletion in exon 5, which could affect its function in dormancy regulation. Japanese plum cultivars are the result of hybridization between *Prunus salicina* and other species (Guerra and Rodrigo 2015) resulting in high genetic diversity and a complex population structure (Guerrero et al. 2021). Since inter-species hybridization is a common phenomenon within the *Prunus* genus (Guerrero et al. 2022), this could explain the discrepancies in *DAM* gene clustering, as these genes may exhibit a combination of characteristics from different species.

### Location- and cultivar-specific gene expression differences during dormancy stages in low- and high-chill-requirement genotypes

The number and distribution of DEGs across cultivars and climates revealed distinct regulatory responses during dormancy and its release. During dormancy (T0), HR showed a higher number of DEGs under Mediterranean subtropical conditions, whereas CG responded more strongly under semi-arid conditions, suggesting cultivar-dependent responses to climatic conditions, likely related to differences in adaptation and chilling requirements. The low number of shared DEGs between climates and cultivars indicates that individual gene-level responses are highly context dependent. At T1, few DEGs were identified within each climate, and none were shared across all cultivar-climate combinations, suggesting that dormancy release does not rely on a single common set of DEGs detectable by this contrast. However, this gene-level specificity should be interpreted together with the co-expression network analysis, which revealed conserved module-level temporal organization across climates. Thus, different cultivar–climate combinations may recruit partly distinct genes while still maintaining coordinated transcriptional programs associated with dormancy progression. After dormancy release (T2), more DEGs were detected under Mediterranean subtropical conditions, suggesting that warmer conditions may induce stronger transcriptional activity. CG showed twice as many DEGs as HR, potentially reflecting differences in how each cultivar adjusts gene expression after dormancy release. Low-chill cultivars such as CG may remain more responsive to environmental signals after dormancy release, whereas high-chill cultivars such as HR may show a more restrained transcriptional response at this stage.

The functional classification of DEGs showed a clear dominance of metabolism-related genes, especially in the full dormancy stage. This suggests that the plant is adjusting its metabolism to focus on basic survival during this early stage (Fadón et al. 2020a). Genes involved in transcription remain active throughout the dormancy period, highlighting the importance of maintaining gene expression even when most cellular processes are slowed down (Fang et al. 2022). The high number of unclassified genes also points to unknown or species-specific functions that are still not well understood. On the other hand, the low presence of genes related to defense, secondary metabolite production, and cell structure suggests reduced plant activity in stress response and growth pathways during dormancy. This differs from previous studies that identified genes associated with stress resistance during the dormancy stage (Yamane et al. 2008; Vimont et al. 2019; Yu et al. 2020; Niu et al. 2024).

## Conclusion

Our study provides a comprehensive transcriptomic analysis of dormancy regulation in two Japanese plum cultivars with contrasting chilling requirements grown under semi-arid and Mediterranean subtropical climatic conditions. Phenological and transcriptomic data showed that environmental conditions modulate dormancy progression, delaying dormancy stages and reshaping gene expression under Mediterranean subtropical conditions. Differential expression analyses revealed genotype- and climate-dependent responses across dormancy stages, whereas co-expression network analysis showed that these heterogeneous gene-level responses are organized into coordinated regulatory modules, with conserved temporal patterns across climates and cultivar-specific differences in module activation. The pronounced transcriptional activity observed under warmer conditions highlights the strong impact of environmental conditions on dormancy regulation. Overall, these findings indicate that dormancy regulation in Japanese plum combines context-dependent gene expression with broader conserved network-level programs. This integrative framework provides a transcriptomic resource for identifying candidate regulatory pathways and supports future breeding and selection of cultivars better adapted to reduced winter chilling and changing climatic scenarios.

## Supplementary Information

Below is the link to the electronic supplementary material.

Additional file 1: Table S1. mRNA-seq sample metadata, including: No., Original_code, Sample Name, Replicate, Location, Cultivar, Time_point, Concentration (ng/ul), Volume (ul), Total amount (ug), RIN, Sample QC Results, number of raw reads (M), number of unique mapped reads (M), and percentage of unique mapped reads as reported by the RSEM summary (XLSX 14 kb)

Additional file 2: Tables S2 - S6. Table S2. Genes (evm.TU identifiers) from the four co-expression modules selected under semi arid climate conditions, corresponding to modules positively and negatively correlated with dormancy stage (time point) and cultivar, ranked according to intramodular connectivity (kWithin). Table S3. Genes (evm.TU identifiers) from the four co expression modules selected under semi arid climate conditions, corresponding to modules positively and negatively correlated with dormancy stage (time point) and cultivar, ranked according to the combined module membership and trait association metric (|kME| × |GS|). Table S4. Genes (evm.TU identifiers) from the four co-expression modules selected under Mediterranean subtropical climate conditions, corresponding to modules positively and negatively correlated with dormancy stage (time point) and cultivar, ranked according to intramodular connectivity (kWithin). Table S5. Genes (evm.TU identifiers) from the four co expression modules selected under Mediterranean subtropical climate conditions, corresponding to modules positively and negatively correlated with dormancy stage (time point) and cultivar, ranked according to the combined module membership and trait association metric (|kME| × |GS|). Table S6. Top three genes per module selected based on kWithin and |kME| × |GS| rankings from modules showing the highest positive and negative correlations under temperate semi-arid and Mediterranean subtropical climate conditions. Columns include module origin, gene name, BLAST annotation, InterPro domains, Gene Ontology (GO) terms and accessions, and GenBank keywords (XLSX 9121 kb)

Additional file 3: Tables S7 - S10. Table S7. DEGs (padj<0.01) at each stage compared to the other two stages in Japanese plum cultivar "Hiromi Red" cultivar under semi-arid climate. The column "Up_Down" indicates whether the gene was found up- or down-regulated and the column "Time_point" indicates the stage (T0, T1 or T2) in which the test was performed. Table S8. DEGs (padj<0.01) between the consecutive dormancy stages in Japanese plum cultivar "Hiromi Red" cultivar under semi-arid climate. The column "Transition" indicates whether the gene was found differentially expressed in the transition from early to mid-stage or from mid to late stage, and the column "Up_Down" indicates whether the gene is up or down regulated in the given transition. Table S9. DEGs (padj<0.01) at each stage compared to the other two stages in Japanese plum cultivar "Crimson Glo" under semi-arid climate. The column "Up_Down" indicates whether the gene was found up- or down-regulated and the column "Time_point" indicates the stage (T0, T1 or T2) in which the test was performed. Table S10. DEGs (padj<0.01) between the consecutive dormancy stages in Japanese plum cultivar "Crimson Glo" cultivar under semi-arid climate. The column "Transition" indicates whether the gene was found differentially expressed in the transition from early to mid-stage or from mid to late stage, and the column "Up_Down" indicates whether the gene is up or down regulated in the given transition (XLS 9663 kb)

Additional file 4: Tables S11 - S12. Table S11. Significantly enriched Cluster of Orthologous Groups (COG) categories (adjusted *P*-value < 0.01) for DEGs identified across all dormancy stages in Japanese plum cultivar "Hiromi Red" under semi-arid climate. The dataset includes both upregulated and downregulated DEGs, without distinction by specific time points or regulation direction. The "COG_category" column indicates the functional classification of each DEG based on the COG database, while the "Description" column provides a brief summary of the associated functional category. Additional columns include the corresponding Gene Ontology (GO) terms, KEGG Orthology (KO) identifiers, and KEGG pathways associated with each DEG. Table S12. Significantly enriched Cluster of Orthologous Groups (COG) categories (adjusted *P*-value < 0.01) for DEGs identified across all dormancy stages in Japanese plum cultivar "Crimson Glo" under semi-arid climate. The dataset includes both upregulated and downregulated DEGs, without distinction by specific time points or regulation direction. The "COG_category" column indicates the functional classification of each DEG based on the COG database, while the "Description" column provides a brief summary of the associated functional category. Additional columns include the corresponding Gene Ontology (GO) terms, KEGG Orthology (KO) identifiers, and KEGG pathways associated with each DEG (XLSX 2487 kb)

Additional file 5: Tables S13 – 16. Table S13. DEGs (padj<0.01) at each stage compared to the other two stages in Japanese plum cultivar "Hiromi Red" under Mediterranean subtropical climate. The column "Up_Down" indicates whether the gene was found up- or down-regulated and the column "Time_point" indicates the stage (T0, T1 or T2) in which the test was performed. Table S14. DEGs (padj<0.01) between the consecutive dormancy stages in Japanese plum cultivar "Hiromi Red" under Mediterranean subtropical climate. The column "Transition" indicates whether the gene was found differentially expressed in the transition from early to mid-stage or from mid to late stage, and the column "Up_Down" indicates whether the gene is up or down regulated in the given transition. Table S15. DEGs (padj<0.01) at each stage compared to the other two stages in Japanese plum cultivar "Crimson Glo" under Mediterranean subtropical climate. The column "Up_Down" indicates whether the gene was found up- or down-regulated and the column "Time_point" indicates the stage (T0, T1 or T2) in which the test was performed. Table S16. DEGs (padj<0.01) between the consecutive dormancy stages in Japanese plum cultivar "Crimson Glo" under Mediterranean subtropical climate. The column "Transition" indicates whether the gene was found differentially expressed in the transition from early to mid-stage or from mid to late stage, and the column "Up_Down" indicates whether the gene is up or down regulated in the given transition (XLSX 6802 kb)

Additional file 6: Tables S17 – S18. Table S17. Significantly enriched Cluster of Orthologous Groups (COG) categories (adjusted *P-*value < 0.01) for DEGs identified across all dormancy stages in Japanese plum cultivar "Hiromi Red" under Mediterranean subtropical climate. The dataset includes both upregulated and downregulated DEGs, without distinction by specific time points or regulation direction. The "COG_category" column indicates the functional classification of each DEG based on the COG database, while the "Description" column provides a brief summary of the associated functional category. Additional columns include the corresponding Gene Ontology (GO) terms, KEGG Orthology (KO) identifiers, and KEGG pathways associated with each DEG. Table S18. Significantly enriched Cluster of Orthologous Groups (COG) categories (adjusted *P-*value < 0.01) for DEGs identified across all dormancy stages in Japanese plum cultivar "Crimson Glo" under Mediterranean subtropical climate. The dataset includes both upregulated and downregulated DEGs, without distinction by specific time points or regulation direction. The "COG_category" column indicates the functional classification of each DEG based on the COG database, while the "Description" column provides a brief summary of the associated functional category. Additional columns include the corresponding Gene Ontology (GO) terms, KEGG Orthology (KO) identifiers, and KEGG pathways associated with each DEG (XLSX 3594 kb)

Additional file 7: Tables S19 – S23. Table S19. List of DEGs (padj<0.01) common to both Japanese plum cultivars "Hiromi Red" and "Crimson Glo" under both semi-arid and Mediterranean subtropical climates at the full dormancy stage (T0). Table S20. List of DEGs (padj<0.01) uniquely identified in Japanese plum cultivar "Hiromi Red" under both semi-arid and Mediterranean subtropical climates at the full dormancy stage (T0). Table S21. List of DEGs (padj<0.01) uniquely identified in Japanese plum cultivar "Crimson Glo" under both semi-arid and Mediterranean subtropical climates at the full dormancy stage (T0). Table S22. List of DEGs (padj<0.01) identified under semi-arid climate conditions for both Japanese plum cultivars "Hiromi Red" and "Crimson Glo" at the full dormancy stage (T0). Table S23. List of DEGs (padj<0.01) identified under Mediterranean subtropical climate conditions for both Japanese plum cultivars "Hiromi Red" and "Crimson Glo" at the full dormancy stage (T0) (XLSX 25 kb)

Additional file 8: Tables S24 – S27. Table S24. List of DEGs (padj<0.01) uniquely identified in Japanese plum cultivar "Hiromi Red" under both semi-arid and Mediterranean subtropical climates at the dormancy release stage (T1). Table S25. List of DEGs (padj<0.01) uniquely identified in Japanese plum cultivar "Crimson Glo" under both semi-arid and Mediterranean subtropical climates at the dormancy release stage (T1). Table S26. List of DEGs (padj<0.01) identified under semi-arid climate conditions for both Japanese plum cultivars "Hiromi Red" and "Crimson Glo" at the dormancy release stage (T1). Table S27. List of DEGs (padj<0.01) identified under Mediterranean subtropical climate conditions for both Japanese plum cultivars "Hiromi Red" and "Crimson Glo" at the dormancy release stage (T1) (XLSX 18 kb)

Additional file 9: Tables S28 – S32. Table S28. List of DEGs (padj<0.01) common to both Japanese plum cultivars "Hiromi Red" and "Crimson Glo" under both semi-arid and Mediterranean subtropical climates at the full recovery stage (T2). Table S29. List of DEGs (padj<0.01) uniquely identified in Japanese plum cultivar "Hiromi Red" under both semi-arid and Mediterranean subtropical climates at the full recovery stage (T2). Table S30. List of DEGs (padj<0.01) uniquely identified in Japanese plum cultivar "Crimson Glo" under both semi-arid and Mediterranean subtropical climates at the full recovery stage (T2). Table S31. List of DEGs (padj<0.01) identified under semi-arid climate conditions for both Japanese plum cultivars "Hiromi Red" and "Crimson Glo" at the full recovery stage (T2). Table S32. List of DEGs (padj<0.01) identified under Mediterranean subtropical climate conditions for both Japanese plum cultivars "Hiromi Red" and "Crimson Glo" at the full recovery stage (T2) (XLSX 34 kb)

Additional file 10: Tables S33 – S35. Table S33. Enriched DEGs (padj<0.001) identified at the full dormancy stage (T0) under both semi-arid and Mediterranean subtropical climate conditions. The column "Combination" specifies the comparison to which each DEG belongs, including DEGs uniquely identified in Japanese plum cultivars "Hiromi Red" or "Crimson Glo," those common to both cultivars, and those specific to a particular climate. The "COG_category" column indicates the functional classification of each DEG based on the Cluster of Orthologous Groups (COG), while the "Description" column provides a brief summary of the associated functional category. Additional columns display the corresponding Gene Ontology (GO) terms, KEGG Orthology (KO) identifiers, and KEGG pathways associated with each DEG. Table S34. Enriched DEGs (padj<0.001) identified at the dormancy release stage (T1) under both semi-arid and Mediterranean subtropical climate conditions. The column "Combination" specifies the comparison to which each DEG belongs, including DEGs uniquely identified in Japanese plum cultivars "Hiromi Red" or "Crimson Glo," and those specific to a particular climate. The "COG_category" column indicates the functional classification of each DEG based on the Cluster of Orthologous Groups (COG), while the "Description" column provides a brief summary of the associated functional category. Additional columns display the corresponding Gene Ontology (GO) terms, KEGG Orthology (KO) identifiers, and KEGG pathways associated with each DEG. Table S35. Enriched DEGs (padj<0.001) identified at the full recovery stage (T2) under both semi-arid and Mediterranean subtropical climate conditions. The column "Combination" specifies the comparison to which each DEG belongs, including DEGs uniquely identified in Japanese plum cultivars "Hiromi Red" or "Crimson Glo," those common to both cultivars, and those specific to a particular climate. The "COG_category" column indicates the functional classification of each DEG based on the Cluster of Orthologous Groups (COG), while the "Description" column provides a brief summary of the associated functional category. Additional columns display the corresponding Gene Ontology (GO) terms, KEGG Orthology (KO) identifiers, and KEGG pathways associated with each DEG (XLSX 839 kb)

Additional file 11: Figure S1. Trees of the Japanese plum cultivars “Hiromi Red” and “Crimson Glo” grown under semi-arid (A, B) and Mediterranean subtropical (C) climatic conditions (TIF 5571 kb)

Additional file 12: Figure S2. Number of aligned reads in each of the three replicates of the flower bud mRNA-seq samples. Read counts are shown in millions (M) (TIF 1183 kb)

Additional file 13: Figure S3. mRNA-seq dataset overview for Japanese plum cultivars "Hiromi Red" (blue) and "Crimson Glo" (red) under semi-arid and Mediterranean subtropical climate conditions across three dormancy stages using variance stabilizing transformation (VST). A) Boxplots, (B) Hierarchical clustering dendrogram and (C) PCA of the mRNA-seq libraries, both showing that replicates are similar with each other. (D) Heatmap showing the relative expression levels of genes of all the stage-specific samples. Only genes with positive variance were included to highlight the most variable genes. Rows represent genes, and columns represent samples grouped by Stage, Cultivar, and Climate. Both genes and samples were clustered using Euclidean distance and the complete linkage method. Full dormancy (T0): light color; dormancy release (T1): normal color, and full recovery (T2): darker color. Semi-arid climate is represented by circles (○), while Mediterranean subtropical climate is represented by triangles (△) (TIF 713 kb)

Additional file 14: Figure S4. Construction of weighted gene co-expression network in samples under semi-arid climate conditions. A) Soft-threshold power selection based on scale-free topology criterion. B) Module size distribution showing the number of genes contained in each module. C) Heatmap of correlations between cultivar and dormancy stage time and module eigengenes. Each column corresponds to a variable and each row corresponds to a module. Cells display Pearson correlation coefficients, where blue indicates negative correlations and red indicates positive correlations. Statistically significant associations (*p* < 0.005) are marked with an asterisk (*) (TIF 9063 kb)

Additional file 15: Figure S5. Construction of weighted gene co-expression network in samples under Mediterranean subtropical climate conditions. A) Soft-threshold power selection based on scale-free topology criterion. B) Module size distribution showing the number of genes contained in each module. C) Heatmap of correlations between cultivar and dormancy stage variables and module eigengenes. Each column corresponds to a variable and each row corresponds to a module. Cells display Pearson correlation coefficients, where blue indicates negative correlations and red indicates positive correlations. Statistically significant associations (*p* < 0.005) are marked with an asterisk (*) (TIF 9063 kb)

Additional file 16: Figure S6. Visualization of selected co expression modules and hub genes under Mediterranean subtropical climate conditions. A–D) Top 20 genes ranked by intramodular connectivity in modules correlated with dormancy stage time point (blue, positive; turquoise, negative) or cultivar (green, positive; yellow, negative). The characteristic expression profile of each module relative to the associated variable is shown. Hub genes (top 3) are highlighted in yellow, and node size is proportional to intramodular connectivity. The annotated hub genes are: blue module (*evm.TU.Chr2.1700, evm.TU.Chr2.1758, evm.TU.Chr7.1416*), turquoise module (*evm.TU.Chr4.1112, evm.TU.Chr4.2779, evm.TU.Chr5.1714*), green module (*evm.TU.Chr2.401, evm.TU.Chr2.614, evm.TU.Chr3.588*), and yellow module (evm.TU.Chr2.956, evm.TU.Chr5.1545, evm.TU.Chr6.1852) (TIF 1240 kb)

Additional file 17: Figure S7. Significantly enriched Cluster of Orthologous Groups (COG) categories (adjusted *P*-value < 0.01) for downregulated and upregulated genes at each dormancy stage in Japanese plum cultivar "Hiromi Red" (A and B, respectively) under semi-arid climate (TIF 7758 kb)

Additional file 18: Figure S8. Significantly enriched Cluster of Orthologous Groups (COG) categories (adjusted *P*-value < 0.01) for downregulated and upregulated genes at each dormancy stage in Japanese plum cultivar "Crimson Glo" (A and B, respectively) under semi-arid climate (TIF 7759 kb)

Additional file 19: Figure S9. Significantly enriched Cluster of Orthologous Groups (COG) categories (adjusted *P*-value < 0.01) for downregulated and upregulated genes at each dormancy stage in Japanese plum cultivar "Hiromi Red" (A and B, respectively) under Mediterranean subtropical climate (TIF 7472 kb)

Additional file 20: Figure S10. Significantly enriched Cluster of Orthologous Groups (COG) categories (adjusted *P*-value < 0.01) for downregulated and upregulated genes at each dormancy stage in Japanese plum cultivar "Crimson Glo" (A and B, respectively) under Mediterranean subtropical climate (TIF 7473 kb)

Additional file 21: Figure S11. Phylogenetic tree of the DAM protein family in* Prunus* species, inferred from Bayesian analysis. Numbers adjacent to the nodes indicate posterior probabilities; only nodes with values greater than 0.7 are labeled. The scale bar represents 0.5 amino acid substitutions per site (TIF 650 kb)

## Data Availability

The raw transcriptome datasets are available in the National Center for Biotechnology Information (NCBI, https:// www. ncbi. nlm. nih. gov/) under the BioProject number PRJNA1276976. The scripts used to process and analyze the data are available at GitHub repository https://github.com/sherlg/transcriptomics-dormancy-japanese-plum.
