# Supplementary figures and images for: Dormancy dynamics in Japanese plum: transcriptomic responses to variable climatic conditions and chilling requirements
Source: Planta. 2026 Aug 1;264(3):78. doi: 10.1007/s00425-026-05104-w (PMC13428783; doi:10.1007/s00425-026-05104-w)

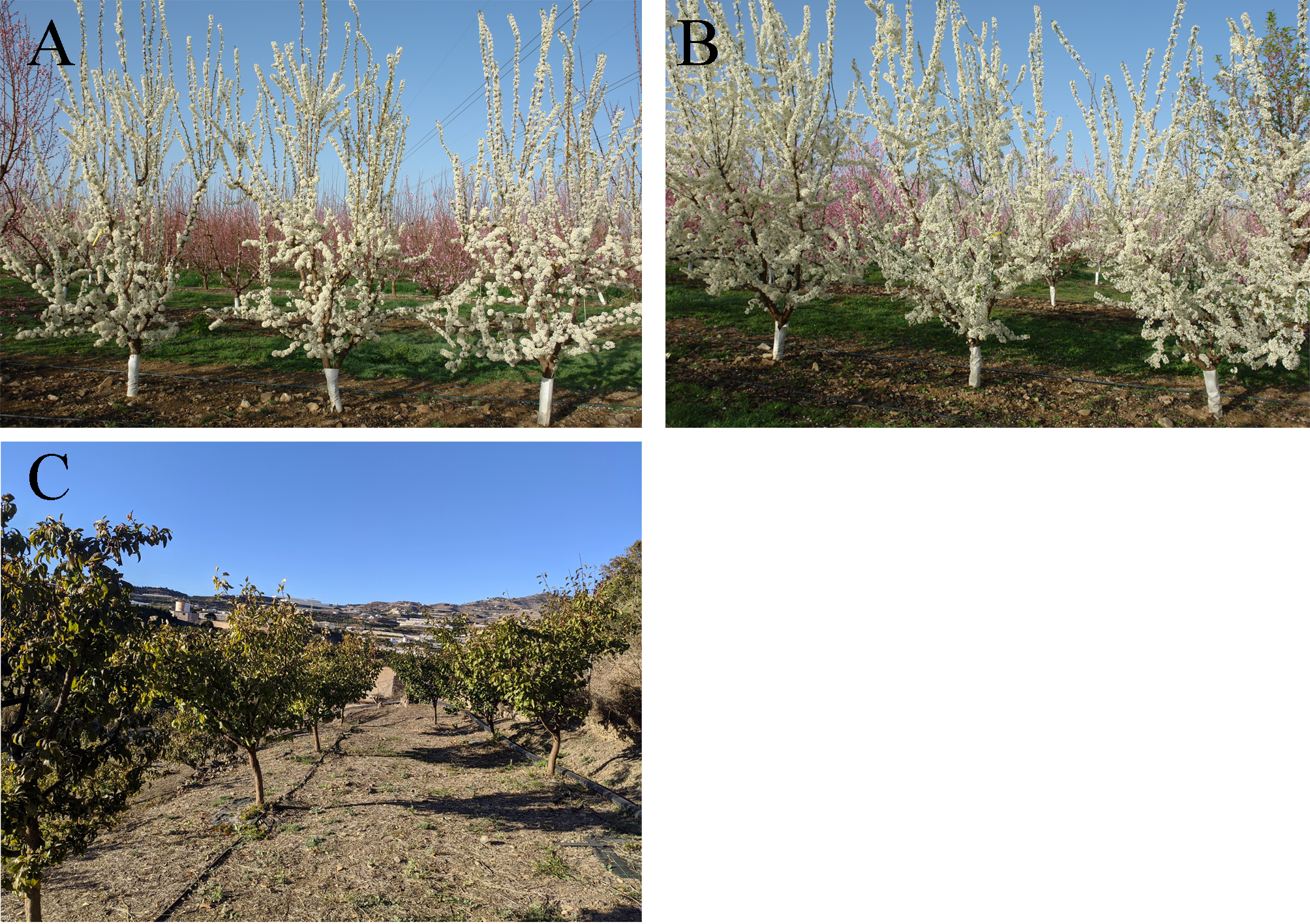

Supplement: Supplementary file 11 — Additional file 11: Figure S1. Trees of the Japanese plum cultivars “Hiromi Red” and “Crimson Glo” grown under semi-arid (A, B) and Mediterranean subtropical (C) climatic conditions (TIF 5571 kb) [file 425_2026_5104_MOESM11_ESM.tif]

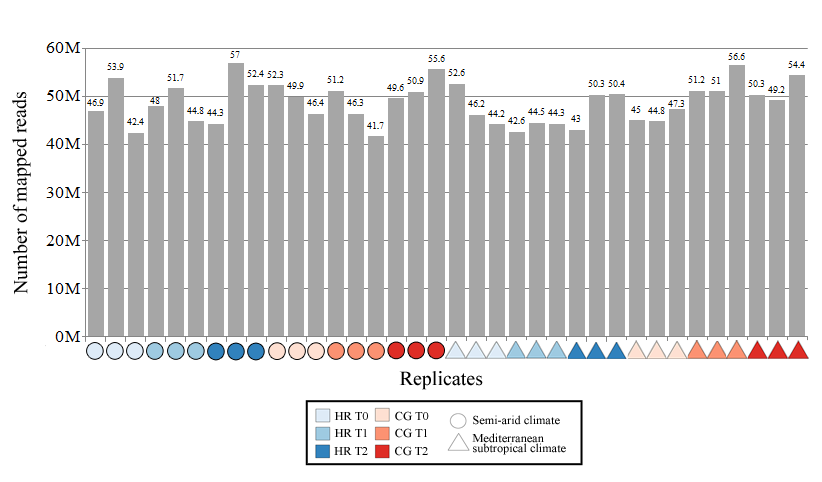

Supplement: Supplementary file 12 — Additional file 12: Figure S2. Number of aligned reads in each of the three replicates of the flower bud mRNA-seq samples. Read counts are shown in millions (M) (TIF 1183 kb) [file 425_2026_5104_MOESM12_ESM.tif]

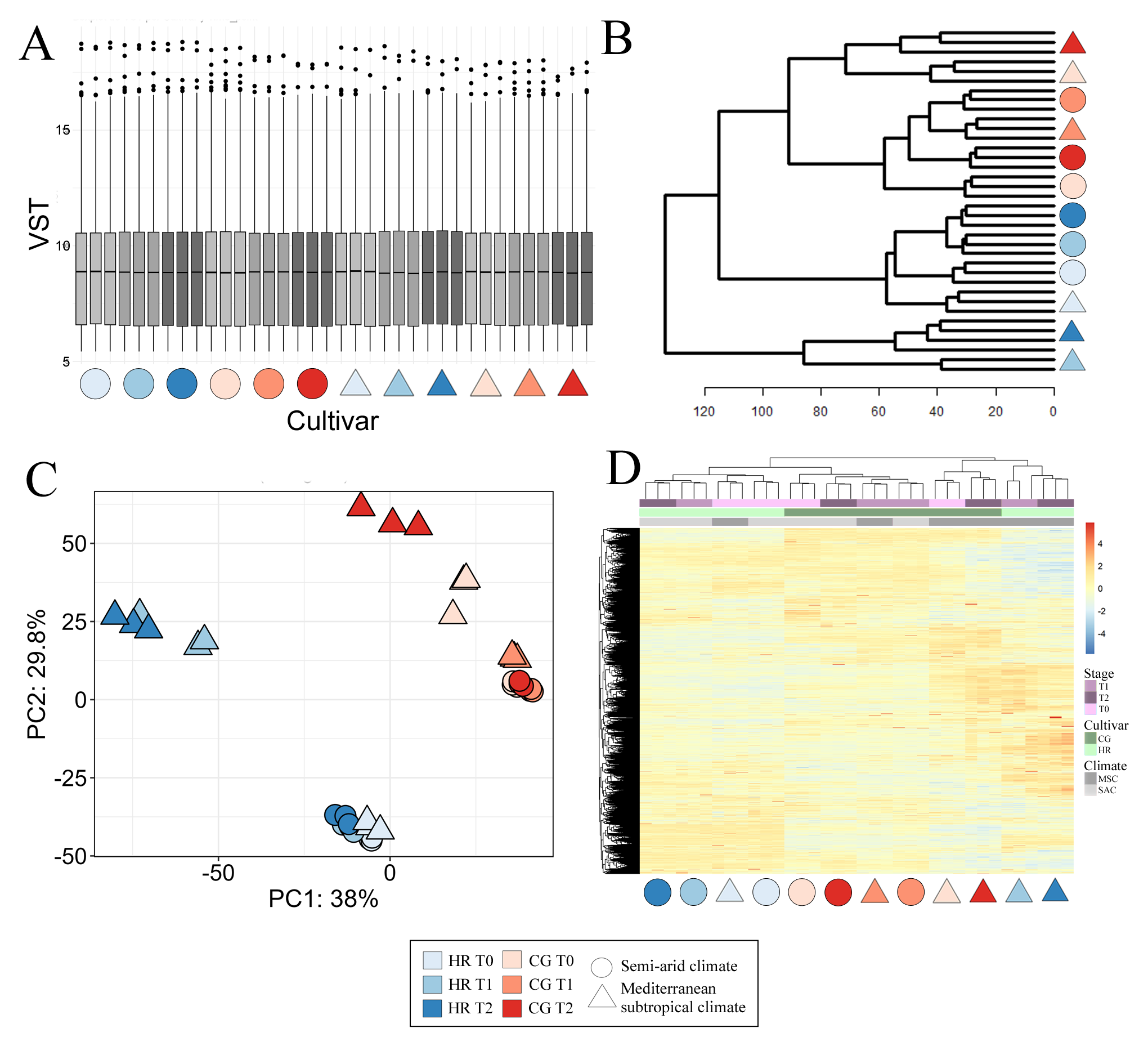

Supplement: Supplementary file 13 — Additional file 13: Figure S3. mRNA-seq dataset overview for Japanese plum cultivars "Hiromi Red" (blue) and "Crimson Glo" (red) under semi-arid and Mediterranean subtropical climate conditions across three dormancy stages using variance stabilizing transformation (VST). A) Boxplots, (B) Hierarchical clustering dendrogram and (C) PCA of the mRNA-seq libraries, both showing that replicates are similar with each other. (D) Heatmap showing the relative expression levels of genes of all the stage-specific samples. Only genes with positive variance were included to highlight the most variable genes. Rows represent genes, and columns represent samples grouped by Stage, Cultivar, and Climate. Both genes and samples were clustered using Euclidean distance and the complete linkage method. Full dormancy (T0): light color; dormancy release (T1): normal color, and full recovery (T2): darker color. Semi-arid climate is represented by circles (○), while Mediterranean subtropical climate is represented by triangles (△) (TIF 713 kb) [file 425_2026_5104_MOESM13_ESM.tif]

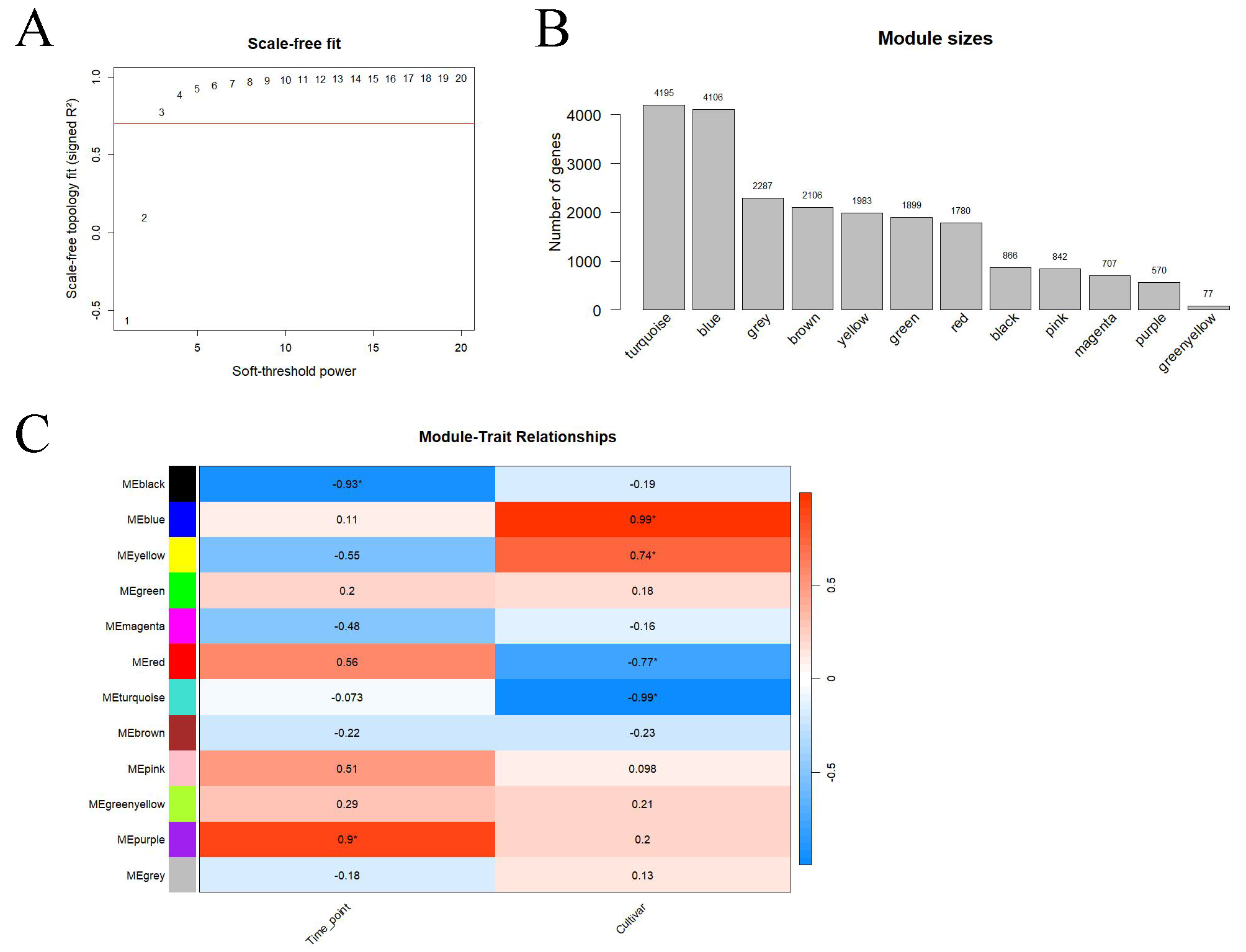

Supplement: Supplementary file 14 — Additional file 14: Figure S4. Construction of weighted gene co-expression network in samples under semi-arid climate conditions. A) Soft-threshold power selection based on scale-free topology criterion. B) Module size distribution showing the number of genes contained in each module. C) Heatmap of correlations between cultivar and dormancy stage time and module eigengenes. Each column corresponds to a variable and each row corresponds to a module. Cells display Pearson correlation coefficients, where blue indicates negative correlations and red indicates positive correlations. Statistically significant associations (p < 0.005) are marked with an asterisk (*) (TIF 9063 kb) [file 425_2026_5104_MOESM14_ESM.tif]

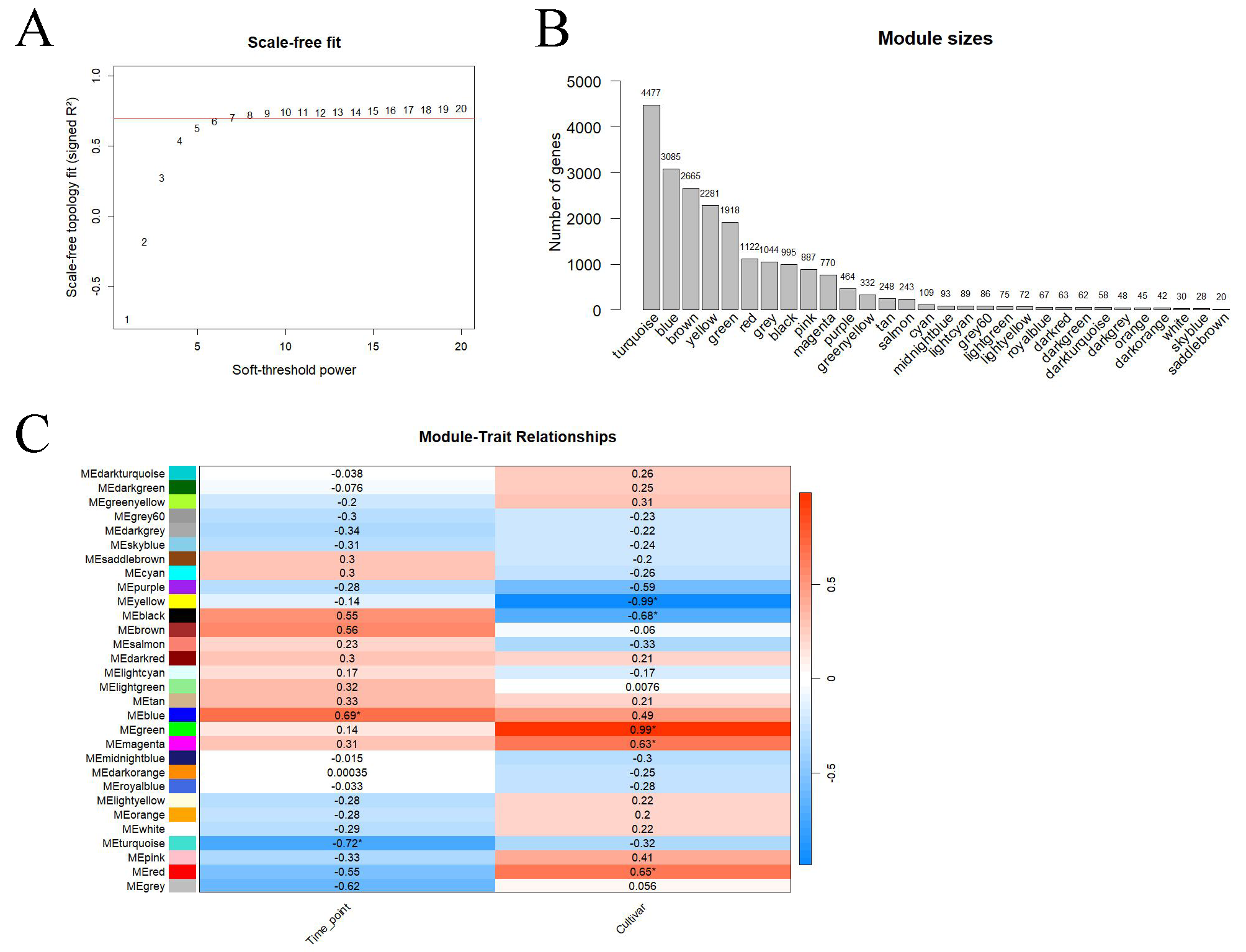

Supplement: Supplementary file 15 — Additional file 15: Figure S5. Construction of weighted gene co-expression network in samples under Mediterranean subtropical climate conditions. A) Soft-threshold power selection based on scale-free topology criterion. B) Module size distribution showing the number of genes contained in each module. C) Heatmap of correlations between cultivar and dormancy stage variables and module eigengenes. Each column corresponds to a variable and each row corresponds to a module. Cells display Pearson correlation coefficients, where blue indicates negative correlations and red indicates positive correlations. Statistically significant associations (p < 0.005) are marked with an asterisk (*) (TIF 9063 kb) [file 425_2026_5104_MOESM15_ESM.tif]

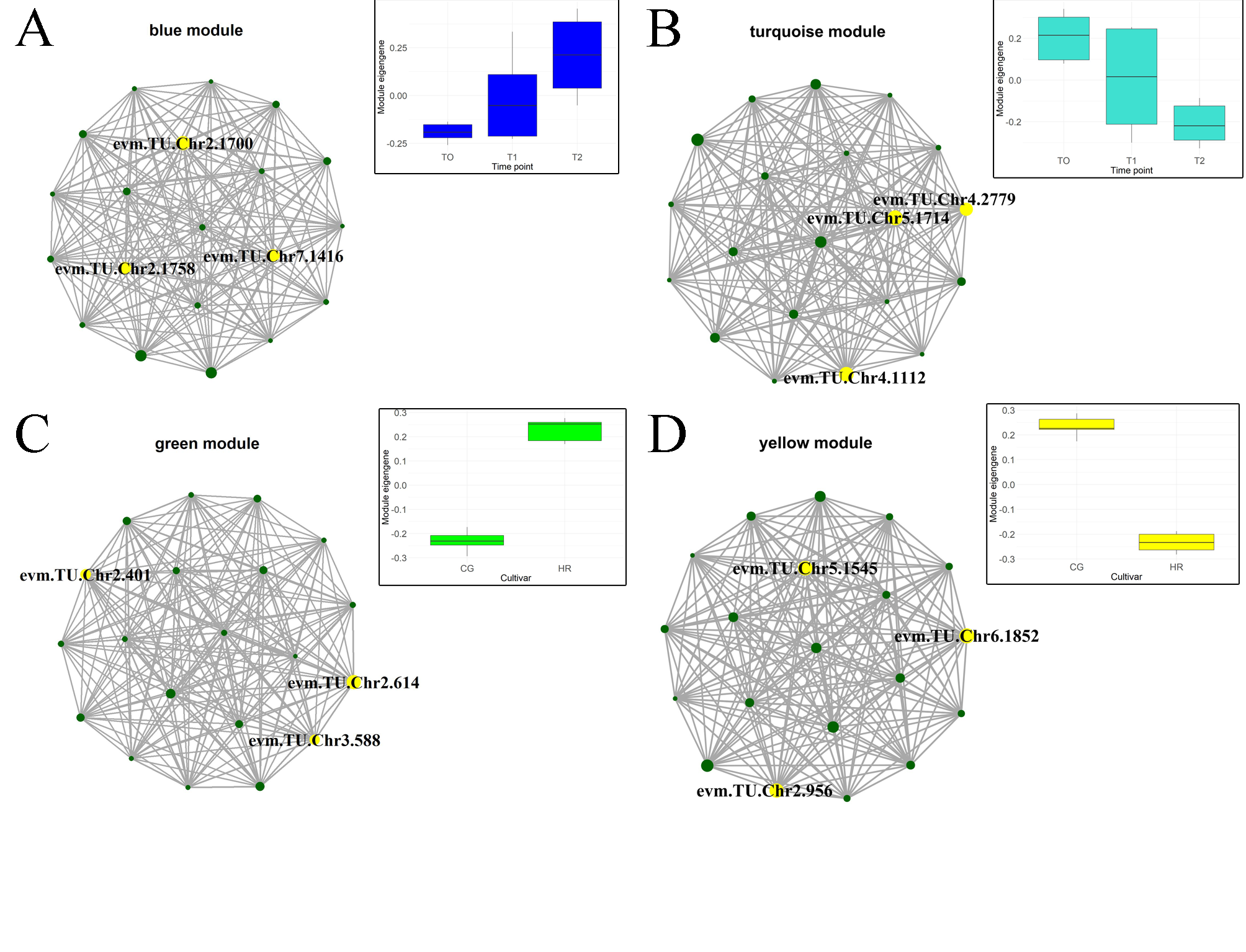

Supplement: Supplementary file 16 — Additional file 16: Figure S6. Visualization of selected co expression modules and hub genes under Mediterranean subtropical climate conditions. A–D) Top 20 genes ranked by intramodular connectivity in modules correlated with dormancy stage time point (blue, positive; turquoise, negative) or cultivar (green, positive; yellow, negative). The characteristic expression profile of each module relative to the associated variable is shown. Hub genes (top 3) are highlighted in yellow, and node size is proportional to intramodular connectivity. The annotated hub genes are: blue module (evm.TU.Chr2.1700, evm.TU.Chr2.1758, evm.TU.Chr7.1416), turquoise module (evm.TU.Chr4.1112, evm.TU.Chr4.2779, evm.TU.Chr5.1714), green module (evm.TU.Chr2.401, evm.TU.Chr2.614, evm.TU.Chr3.588), and yellow module (evm.TU.Chr2.956, evm.TU.Chr5.1545, evm.TU.Chr6.1852) (TIF 1240 kb) [file 425_2026_5104_MOESM16_ESM.tif]

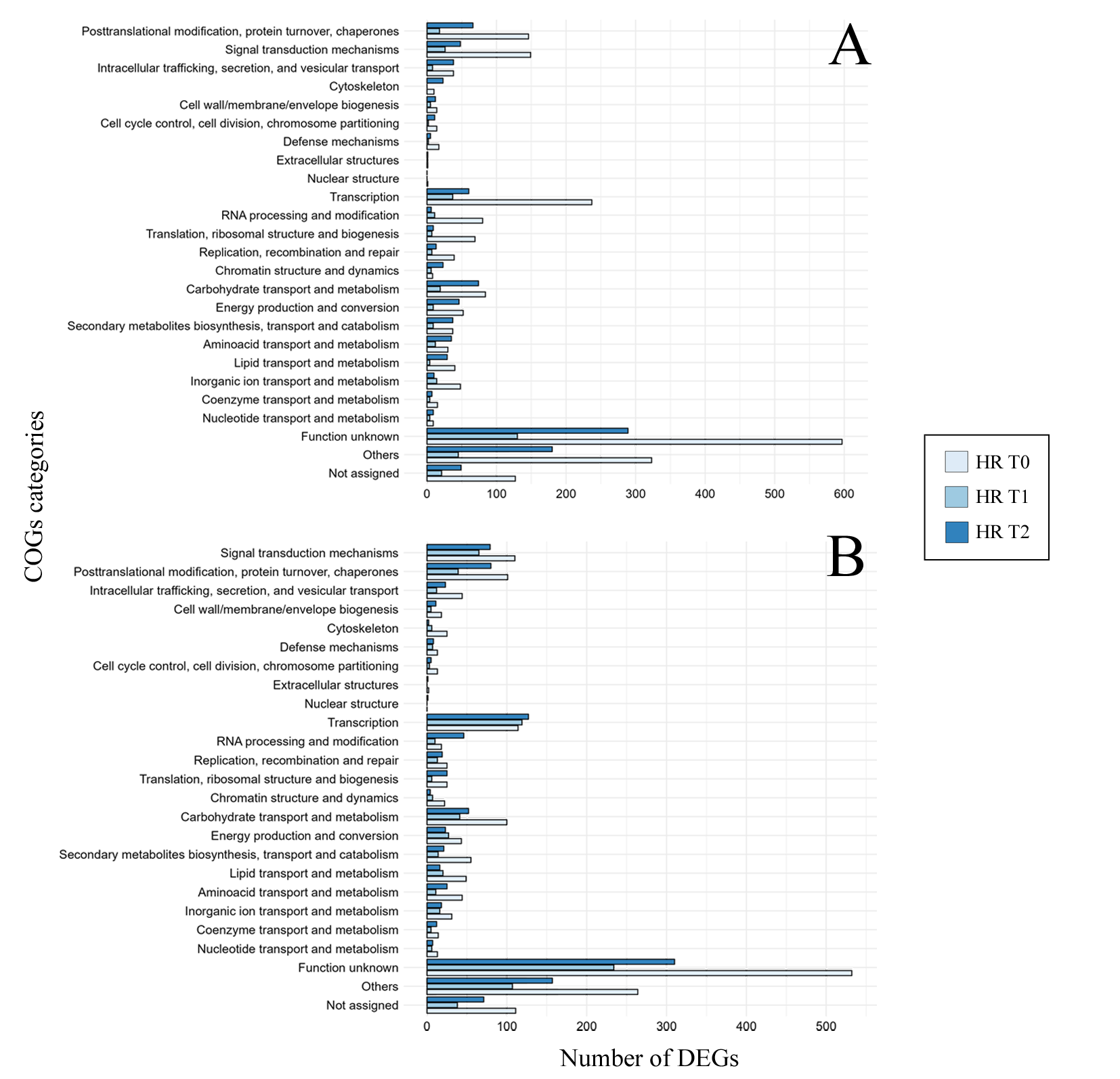

Supplement: Supplementary file 17 — Additional file 17: Figure S7. Significantly enriched Cluster of Orthologous Groups (COG) categories (adjusted P-value < 0.01) for downregulated and upregulated genes at each dormancy stage in Japanese plum cultivar "Hiromi Red" (A and B, respectively) under semi-arid climate (TIF 7758 kb) [file 425_2026_5104_MOESM17_ESM.tif]

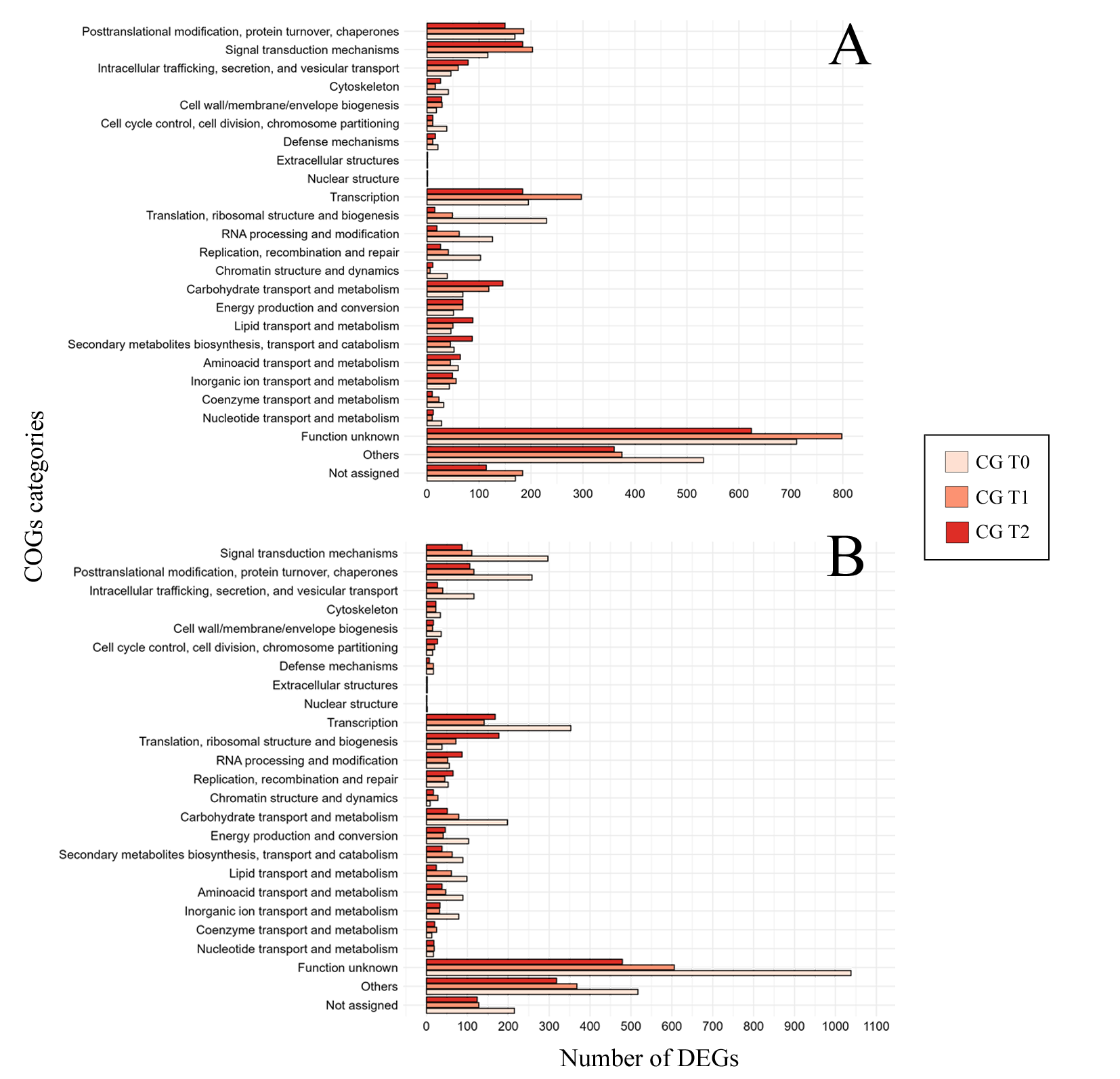

Supplement: Supplementary file 18 — Additional file 18: Figure S8. Significantly enriched Cluster of Orthologous Groups (COG) categories (adjusted P-value < 0.01) for downregulated and upregulated genes at each dormancy stage in Japanese plum cultivar "Crimson Glo" (A and B, respectively) under semi-arid climate (TIF 7759 kb) [file 425_2026_5104_MOESM18_ESM.tif]

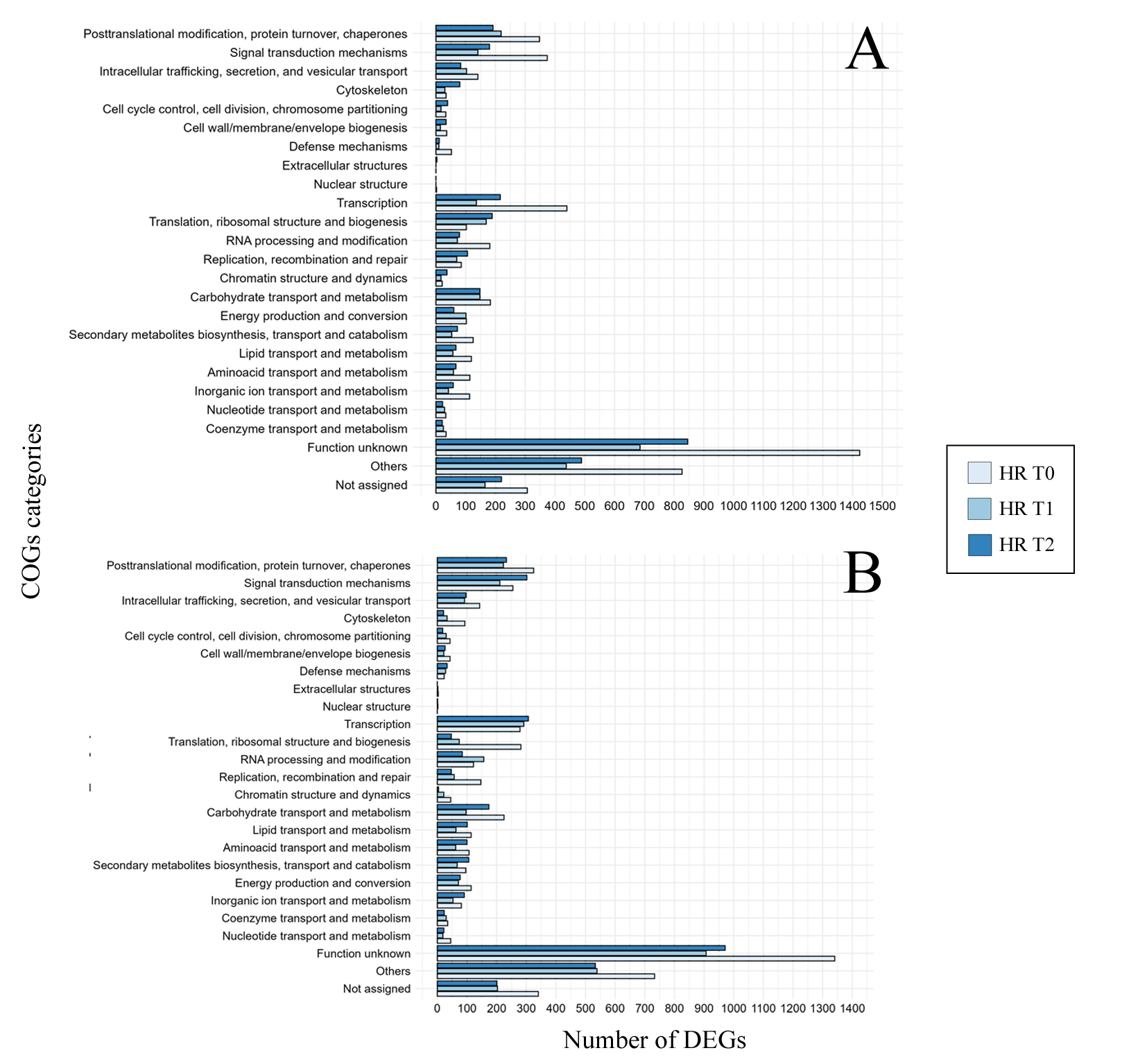

Supplement: Supplementary file 19 — Additional file 19: Figure S9. Significantly enriched Cluster of Orthologous Groups (COG) categories (adjusted P-value < 0.01) for downregulated and upregulated genes at each dormancy stage in Japanese plum cultivar "Hiromi Red" (A and B, respectively) under Mediterranean subtropical climate (TIF 7472 kb) [file 425_2026_5104_MOESM19_ESM.tif]

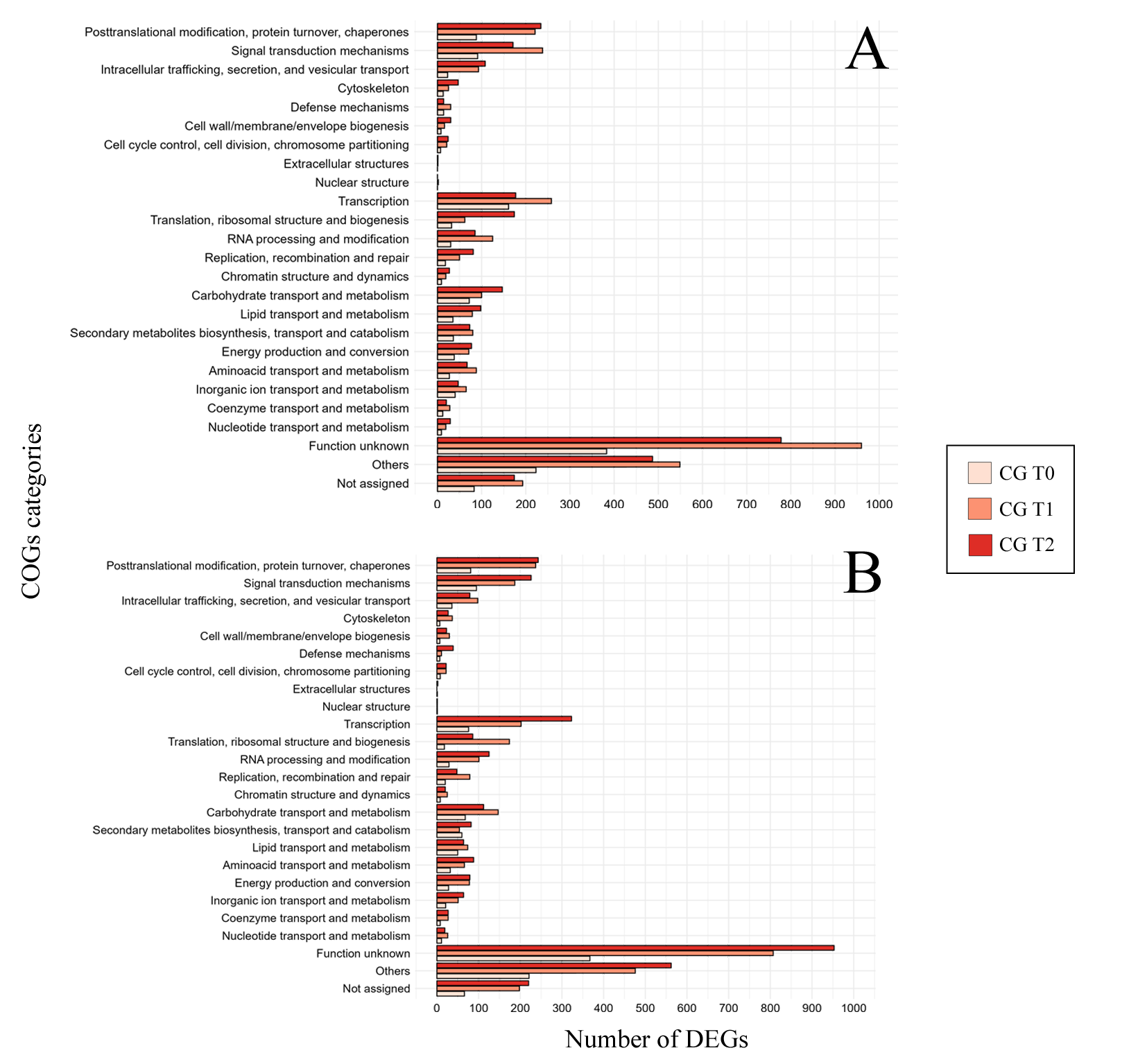

Supplement: Supplementary file 20 — Additional file 20: Figure S10. Significantly enriched Cluster of Orthologous Groups (COG) categories (adjusted P-value < 0.01) for downregulated and upregulated genes at each dormancy stage in Japanese plum cultivar "Crimson Glo" (A and B, respectively) under Mediterranean subtropical climate (TIF 7473 kb) [file 425_2026_5104_MOESM20_ESM.tif]

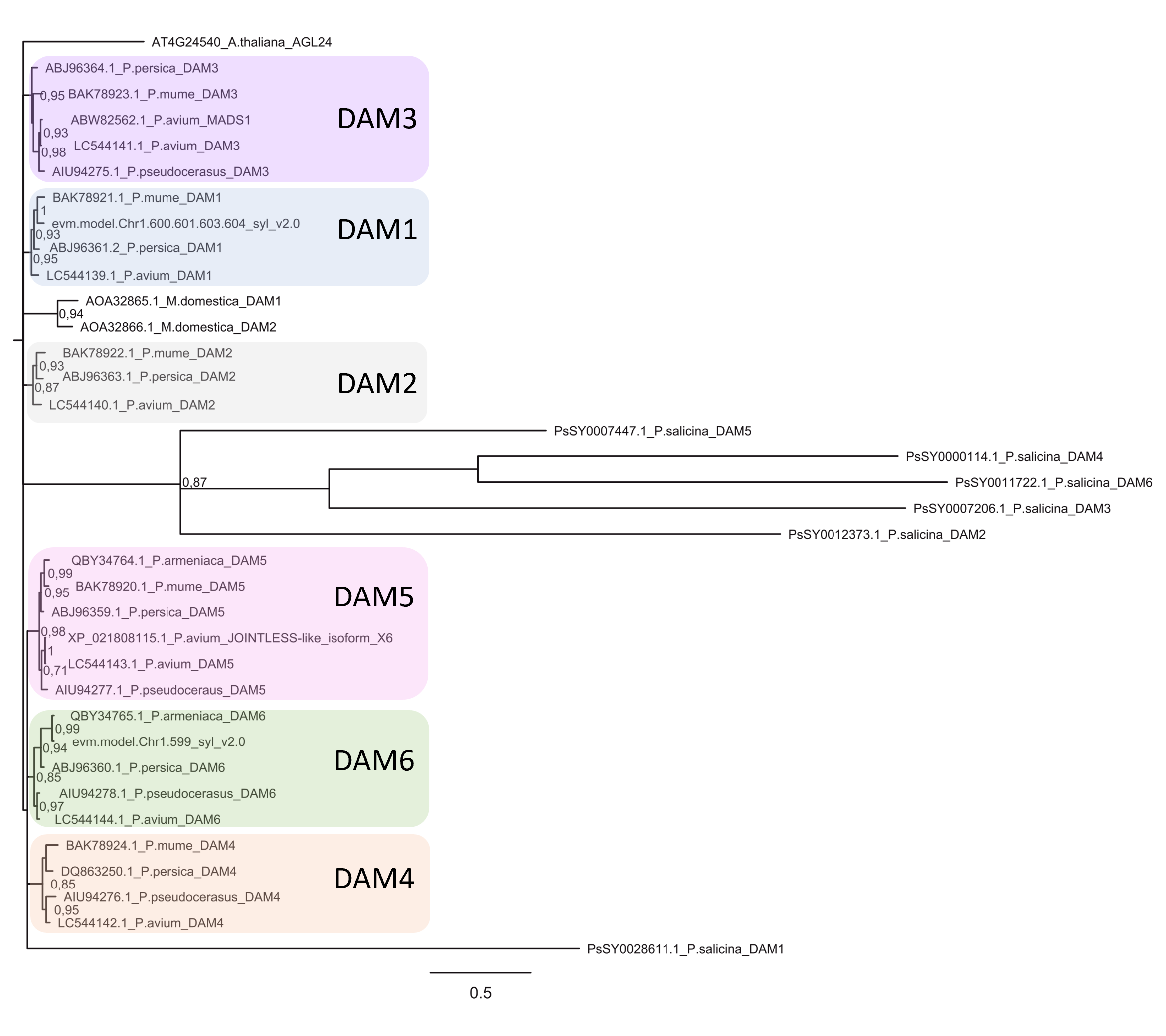

Supplement: Supplementary file 21 — Additional file 21: Figure S11. Phylogenetic tree of the DAM protein family in Prunus species, inferred from Bayesian analysis. Numbers adjacent to the nodes indicate posterior probabilities; only nodes with values greater than 0.7 are labeled. The scale bar represents 0.5 amino acid substitutions per site (TIF 650 kb) [file 425_2026_5104_MOESM21_ESM.tif]
